# Epigenetic dysregulation in aged muscle stem cells drives mesenchymal progenitor expansion via IL-6 and Spp1 signaling

**DOI:** 10.1038/s43587-025-01002-0

**Published:** 2025-10-29

**Authors:** Giulia Riparini, Morgan Mackenzie, Faiza Naz, Stephen Brooks, Kan Jiang, Anshu Deewan, Brittany Dulek, Shamima Islam, Kyung Dae Ko, Wanxia L. Tsai, Massimo Gadina, Stefania Dell’Orso, Vittorio Sartorelli

**Affiliations:** 1 Laboratory of Muscle Stem Cells and Gene Regulation, National Institute of Arthritis and Musculoskeletal and Skin Diseases, National Institutes of Health, Bethesda, MD USA; 2 Genomic Technology Section, National Institute of Arthritis and Musculoskeletal and Skin Diseases, National Institutes of Health, Bethesda, MD USA; 3 Biodata Mining and Discovery Section, National Institute of Arthritis and Musculoskeletal and Skin Diseases, National Institutes of Health, Bethesda, MD USA; 4 Integrated Data Sciences Section, Research Technologies Branch, National Institute of Allergy and Infectious Diseases, National Institutes of Health, Bethesda, MD USA; 5 Translational Immunology Section, National Institute of Arthritis and Musculoskeletal and Skin Diseases, National Institutes of Health, Bethesda, MD USA

**Keywords:** Ageing, Muscle stem cells

## Abstract

Sarcopenia, the age-related decline in muscle mass, strength and function, is characterized by impaired muscle homeostasis, reduced regenerative potential of muscle stem cells (MuSCs) and increased fibrosis. Here we report that aged MuSCs can autonomously instruct fibro-adipogenic progenitors (FAPs) to proliferate and acquire a fibrogenic phenotype, independent of other cell types. Both the polycomb-deficient *Ezh2*^−/−^ mouse model and aged mice exhibited defective regeneration, FAP expansion, fibrosis and elevated secretion of interleukin 6 (IL-6) and secreted phosphoprotein 1 (Spp1; osteopontin) by MuSCs. In aged MuSCs, reduction of the histone H3K27me3 repressive mark at the *Nfbk1* gene correlated with its increased expression and enhanced chromatin recruitment to the *IL6* and *Spp1* genes, leading to their activation. Pharmacological inhibition of IL-6 and Spp1 signaling in co-culture systems or in aged mice reduced FAP proliferation and muscle fibrosis. These findings indicate that epigenetic dysregulation of aged MuSCs contributes to aged-related muscle fibrosis.

## Abstract (web summary)

Riparini et al. demonstrate that aged muscle stem cells amplify fibrosis by expanding mesenchymal progenitors via IL-6 and Spp1. Blocking these signals in aged mice curbs fibrosis and enhances muscle repair.

## Main

MuSCs maintain homeostasis and are essential for regeneration^1–4^. Reduced number and impaired function of MuSCs, along with the the progressive accumulation of fibrotic tissue, are key features of sarcopenia—the loss of muscle mass and strength that occurs with aging^5–7^. Aged MuSCs exhibit signs of intrinsic dysregulation, including the inappropriate activation of a pro-fibrotic genetic program^8^. Moreover, inflammatory and pro-fibrotic signals from circulating and muscle-resident immune and mesenchymal cells negatively impact MuSC function, leading to increased extracellular matrix (ECM) deposition^9–12^. Upon injury or increased load, muscle-resident mesenchymal cells, known as FAPs, initially expand to support MuSC proliferation and differentiation^13^. However, their number later declines due to apoptosis induced by macrophage-produced tumor necrosis factor (TNF)^14^. In pathological conditions and aging, FAP contraction is disrupted, resulting in abnormal persistence, expansion and differentiation of FAPs into specific mesenchymal cell lineages^9,10,15,16^. The mechanisms underlying this phenomenon are not fully understood.

Deletion of the polycomb repressive complex 2 (PRC2) subunit *Ezh2*, which encodes the enzyme that deposits the repressive histone mark H3K27me3, recapituales aspects of aging in various cell types^17,18^. Furthermore, MuSCs from young mice with constitutive *Ezh2* deletion, as well as injured aged skeletal muscle and geriatric MuSCs, all show upregulated expression of the senescent marker *p16*^*INK4*^ (*Cdkn2a*)^19,20^. These observations prompted us to inducibly delete Ezh2 in adult MuSCs (i*Ezh2*^−/−^ mice) as a potential model for mimicking certain aspects of aging. The i*Ezh2*^−/−^ mice showed defective MuSC proliferation and muscle regeneration, along with expansion of FAPs and persistent fibrosis. Similarly, aged mice showed characteristics akin to i*Ezh2*^−/−^ mice, including impaired MuSC proliferation and poor regeneration, FAP expansion and increased fibrosis. Dysregulated expression of IL-6 and Spp1 (osteopontin) in i*Ezh2*^−/−^ triggered the proliferation of fibrogenic FAPs. Aged MuSCs also expressed and secreted higher levels of IL-6 and Spp1 compared to adult MuSCs, further promoting the expansion of fibrogenic FAPs. Erosion of H3K27me3 at the *Nfkb1* gene in aged MuSCs was associated with increased expression of NF-κB, its chromatin recruitment and activation of the *IL6* and *Spp1* genes. Reducing NF-κB levels decreased IL-6 and Spp1 in aged MuSCs. Co-cultured aged MuSCs stimulated the proliferation of fibrogenic FAPs, and blocking IL-6 and Spp1 signaling in co-cultures or aged mice reduced FAP proliferation and muscle fibrosis. Altogether, our findings support the functional role of epigenetic changes in aged MuSCs in driving signaling pathways that promote fibrosis and provide insights into potential therapeutic strategies for age-related muscle fibrosis.

## Results

### Deletion of the polycomb methyltransferase *Ezh2* in adult MuSCs results in impaired muscle regeneration and fibrosis

**Fig. 1:**
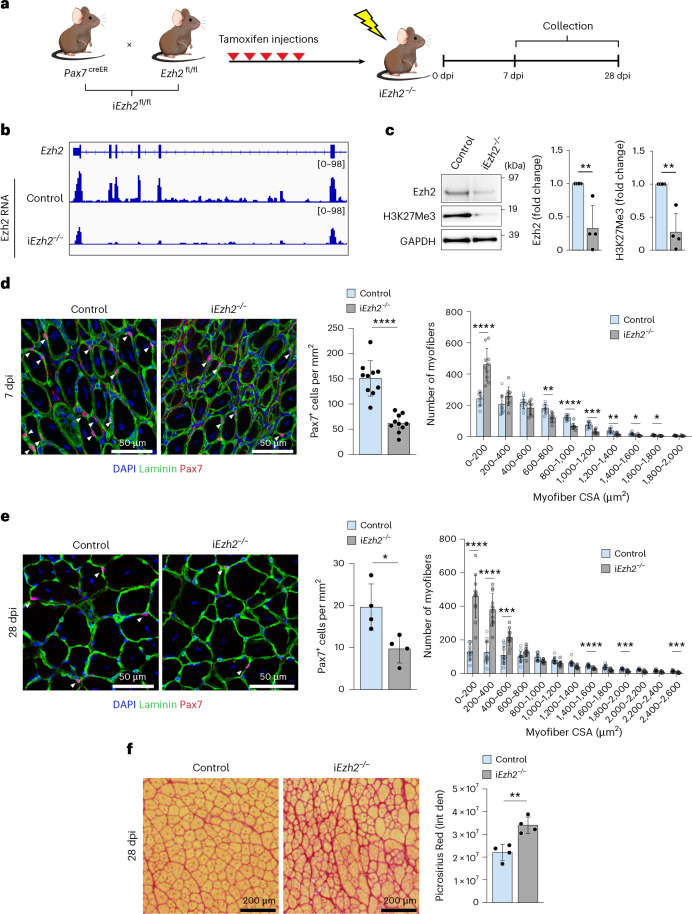
Deletion of the polycomb methyltransferase *Ezh2* in adult MuSCs results in impaired muscle regeneration and fibrosis. **a**, Breeding schematic of i*Ezh2* mice, including tamoxifen treatment and time of sample collection. **b**, RNA-seq tracks showing Ezh2 expression in control and i*Ezh2*^−/−^ MuSCs at 7 dpi. **c**, Immunoblot and quantification of Ezh2 and H3K27me3 protein levels in MuSCs at 7 dpi. GAPDH antibody was used as a loading control. Data are presented as mean ± s.d., two-tailed unpaired *t*-test, *n* = 4 mice, ^**^P = 0.0079 (Ezh2), ^**^P = 0.0023 (H3K27me3). **d**,**e**, Laminin and Pax7 immunostaining of TA muscle sections from control or i*Ezh2*^−/−^ mice at 7 dpi (**d**) and 28 dpi (**e**). DAPI stains nuclei. Quantification of Pax7^+^ cells and myofiber CSA in control or i*Ezh2*^−/−^ mice at 7 dpi (**d**) and 28 dpi (**e**). At least two muscle sections per mouse were analyzed. Data are presented as mean ± s.d., two-tailed unpaired *t*-test. **d**, *n* = 10 mice for Pax7 quantification, ^****^P < 0.0001; *n* = 10 from five control or i*Ezh2*^−/−^ mice, respectively, for CSA analysis, ^*^P = 0.0273 (1,400−1,600-μm^2^ fibers), ^*^P = 0.0353 (1,600−1,800-μm^2^ fibers), ^**^P = 0.0011 (600−800-μm^2^ fibers), ^**^P = 0.0033 (1,200−1,400-μm^2^ fibers), ^***^P = 0.0001 (1,000−1,200-μm^2^ fibers), ^****^P < 0.0001 (0−200-μm^2^ fibers; 800−1,000-μm^2^ fibers). **e**, *n* = 4 mice for Pax7 quantification, ^*^P = 0.0224; *n* = 12 from five control or i*Ezh2*^−/−^ mice, respectively, for CSA analysis, ^***^P < 0.0007 (400−600-μm^2^ fibers; 1,800−2,000 μm^2^; 2,400−2,600 μm^2^ fibers), ^****^P < 0.0001 (0−200 μm^2^; 200−400-μm^2^ fibers; 1,400−1,600-μm^2^ fibers). **f**, Picrosirius Red staining and quantification of fibrotic area on TA muscle sections from control and i*Ezh2*^−/−^ mice at 28 dpi. At least two muscle sections per mouse were analyzed. Data are presented as mean ± s.d., two-tailed unpaired *t*-test, *n* = 4 mice, ^**^P = 0.0032. Source data

**Extended Data Fig. 1:**
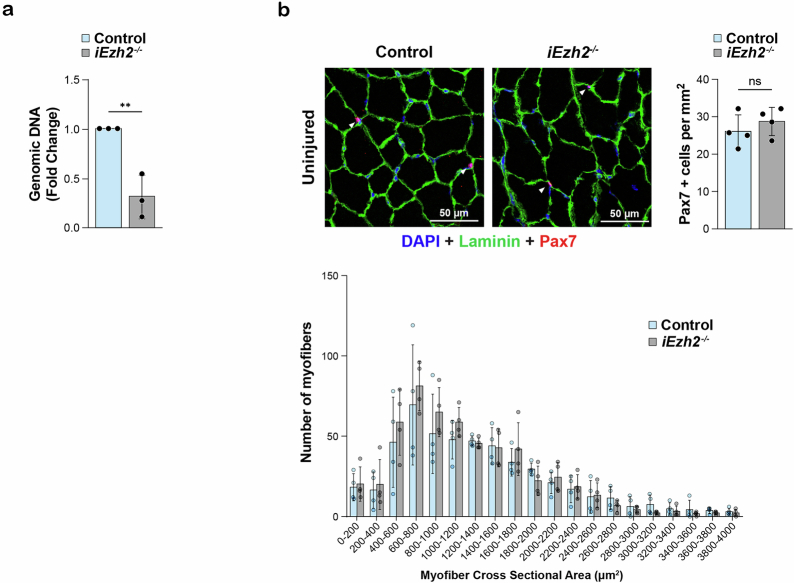
Uninjured i*Ezh2*^−/−^ mice do not display a phenotype. **a**, *Ezh2* deletion in i*Ezh2*^−/−^ mice. Genomic DNA was isolated from control or i*Ezh2*^−/−^ mice, and qPCR was performed with primers spanning the *Ezh2* gene. Data are represented as mean ± SD (n = 3 mice) ^**^ P = 0.0057. **b**, Laminin and Pax7 immunostaining of TA muscle sections from uninjured control or i*Ezh2*^−/−^ mice. Quantification of Pax7^+^ cells and myofiber CSA. Data are presented as mean ± SD (n = 4 mice), ns= non-significant. Two-tailed unpaired *t*-test was used (**a, b**). Source data

Constitutive ablation of PRC2 H3K27 methyltransferase *Ezh2* in MuSCs affects their proliferation and lineage commitment^19,20^. To differentiate the effects of *Ezh2* deletion during development from those in adult MuSCs, we generated a tamoxifen-inducible mouse model. Knock-in mice with tamoxifen-inducible CreER^T2^ recombinase from the Pax7 locus^21^ (*Pax7*^*creER*^) were crossed with mice bearing floxed *Ezh2* alleles (*Ezh2*^*fl/fl*^)^22^, resulting in Tm*Pax7*^*creER*^;*Ezh2*^*fl/fl*^ (inducible i*Ezh2*^*fl/fl*^) mice (Fig. 1a). Tamoxifen injections in 3-month-old mice led to approximately 75% deletion of *Ezh2* alleles (Extended Data Fig. 1a). At this stage, approximately 97% of MuSCs have become quiescent. Unlike constitutive *Pax7*^*cre*^*;Ezh2*^*fl/fl*^ mice^19^, i*Ezh2*^−/−^ mice had no appreciable difference in MuSC number or muscle cross-sectional area (CSA) in homeostatic uninjured conditions (Extended Data Fig. 1b). We performed RNA sequencing (RNA-seq) on fluorescence-activated cell sorting (FACS)-isolated MuSCs from uninjured (basal conditions), tamoxifen-treated adult (3-month-old) control and i*Ezh2*^*fl/fl*^ mice and assessed expression of senescence-associated markers. Of the 80 senescence-associated markers examined^23,24^, only *Ccl8*, *CXCL2* and *IL11* were upregulated in i*Ezh2*^−/−^ MuSCs (Supplementary Table 1). Ezh2 is not expressed in quiescent MuSCs, and, thus, its deletion is not expected to influence gene expression in basal conditions^19,25^. The induction of these three genes in i*Ezh2*^−/−^ MuSCs is likely attributable to initial cell activation caused by muscle dissociation and FACS isolation^26,27^, potentially coupled to their low threshold for Ezh2-dependent transcriptional derepression. The absence of a comprehensive senescent signature in uninjured i*Ezh2*^−/−^ mice mirrors the lack of detectable senescence-associated beta-galactosidase (SA-β-gal)-positive cells in uninjured muscle of geriatric mice^23^. To examine the role of Ezh2 in MuSC activation, tibialis anterior (TA) muscles from control and i*Ezh2*^−/−^ mice were injured with notexin (NTX) and collected at 7 or 28 days post-injury (dpi) (Fig. 1a). At 7 dpi, Ezh2 mRNA, protein and H3K27me3 mark were reduced in FACS-isolated i*Ezh2*^−/−^ MuSCs (Fig. 1b,c). i*Ezh2*^−/−^ mice showed an approximately 65% decrease in MuSC number and a significant reduction of muscle CSA (Fig. 1d). Impaired MuSC proliferation was previously observed in constitutive *Pax7*^cre^;*Ezh2*^fl/fl^ mice^19,20^. MuSCs and CSA remained reduced, and fibrosis was increased at 28 dpi (Fig. 1e,f). Thus, *Ezh2* ablation in adult MuSCs impairs muscle regeneration and leads to increased fibrosis in i*Ezh2*^−/−^ mice.

### Impaired muscle regeneration results in the expansion of FAPs in i*Ezh2*^−/−^ mice

**Fig. 2:**
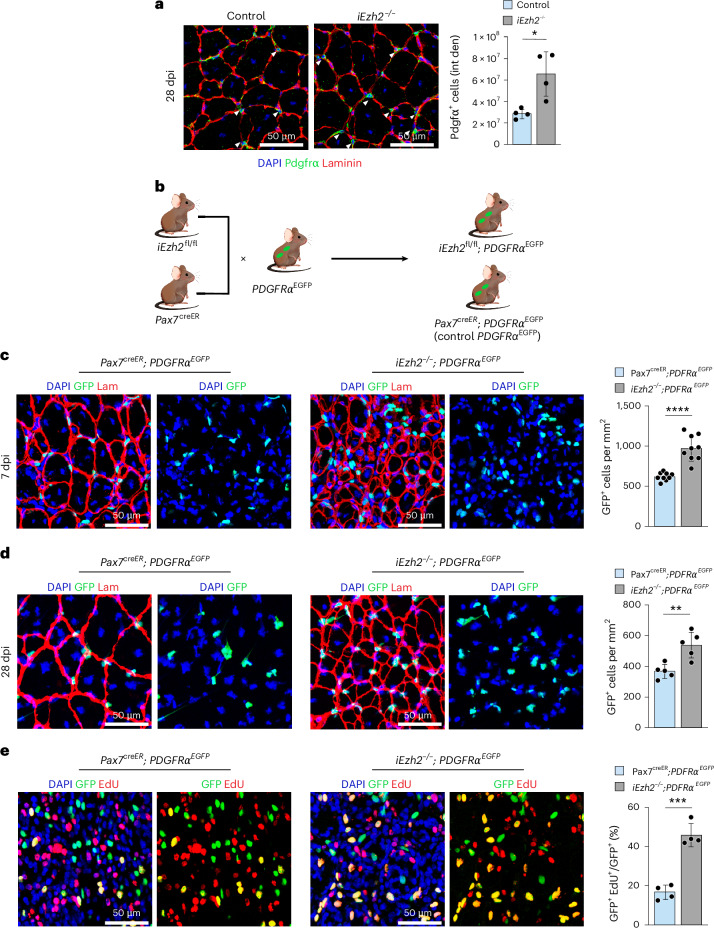
Impaired muscle regeneration results in FAP expansion in i*Ezh2*^−/−^ mice. **a**, Pdgfrα and Laminin immunostaining of TA muscle sections from control and i*Ezh2*^−/−^ mice at 28 dpi. DAPI marks nuclei. Quantification of Pdgfrα^+^ cells is shown on the right. Two muscle sections per mouse were analyzed. Data are presented as mean ± s.d., *n* = 4 mice, ^*^P = 0.0129. **b**, Breeding schematic for *Pax7*^*creER*^ and i*Ezh2*^*fl/fl*^ mice crossed with *PDGFRα*^*EGFP*^ mice. **c**,**d**, Laminin immunostaining of TA muscle sections from control *PDGFRα*^*EGFP*^ or i*Ezh2*^−/−^;*PDGFRα*^*EGFP*^ mice at 7 dpi (**c**) and 28 dpi (**d**). GFP^+^ nuclei identify FAPs; DAPI marks nuclei. Quantification of GFP^+^ cells is shown on the right for each timepoint (**c**,**d**). Two muscle sections per mouse were analyzed. Data are presented as mean ± s.d. **c**, *n* = 9 mice, ^****^P < 0.0001; **d**, *n* = 5 mice, ^**^P = 0.004. **e**, EdU immunostaining of TA muscle sections from control *PDGFRα*^*EGFP*^ or i*Ezh2*^−/−^;*PDGFRα*^*EGFP*^ mice at 4 dpi. GFP^+^ nuclei identify FAPs. Quantification of GFP^+^EdU^+^ cells is shown on the right. Two muscle sections per mouse were analyzed. Data are presented as mean ± s.d., *n* = 4 mice,^***^P = 0.0002. Two-tailed unpaired *t*-test was used for statistical comparisons in **a** and **c**−**e**. Lam, Laminin. Source data

**Extended Data Fig. 2:**
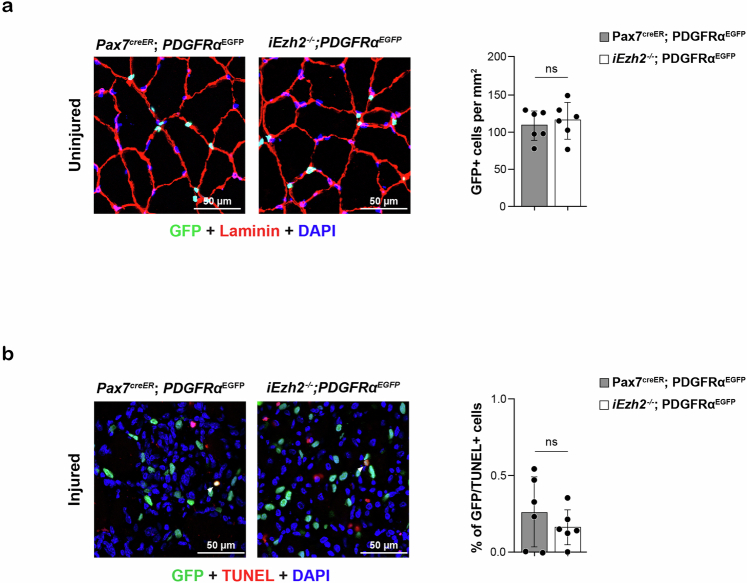
PDGFRa^EGFP+^ cells in uninjured control and i*Ezh2*^−/−^ mice. **a**, Laminin immunostaining and GFP reactivity in TA muscle sections from uninjured control *PDGFRα*^*EGFP*^ or i*Ezh2*^−/−^;*PDGFRα*^*EGFP*^ mice. On the right, quantification of GFP^+^ cells. Data are represented as mean ± SD (n = 6 mice), ns= non-significant. **b**, GFP^+^ and TUNEL^+^ cells in TA muscle sections obtained from uninjured control *PDGFRα*^*EGFP*^ or i*Ezh2*^−/−^;*PDGFRα*^*EGFP*^ mice. Quantification of GFP^+^/TUNEL^+^ cells. Data are presented as mean ± SD (n = 6 mice), ns= non-significant. Two-tailed unpaired *t*-test was used (**a, b**). Source data

FAPs are mesenchymal cells residing in the interstitium of skeletal muscle and other tissues. They are involved in the formation of ECM and are the primary collagen-producing cells in adult muscle tissue, contributing to fibrosis in chronic muscle injuries and muscular dystrophies^9,13,21,28,29^. Confocal microscopy of injured TA muscle sections of control and i*Ezh2*^−/−^ mice immunostained with antibodies directed against the FAP marker platelet-derived growth factor receptor alpha (PDGFRα)^9,13^ revealed an increased number of FAPs in i*Ezh2*^−/−^ mice compared to littermate controls at 28 dpi (Fig. 2a). To confirm this observation, we crossed Pax7^creER^ or i*Ezh2*^−/−^ with *PDGFRα*^*EGFP*^ mice to generate Pax7^creER^;*PDGFRα*^*EGFP*^ (control *PDGFRα*^*EGFP*^) or i*Ezh2*^−/−^;*PDGFRα*^*EGFP*^ mice, respectively (Fig. 2b). In these mice, the nuclear histone H2B−eGFP fusion gene is expressed from the PDGFRα locus, thereby labeling *PDGFRα*-expressing cells. The number of GFP^+^ cells in uninjured control *PDGFRα*^*EGFP*^ or i*Ezh2*^−/−^;*PDGFRα*^*EGFP*^ mice was similar (Extended Data Fig. 2a). By contrast, analysis of 7-dpi injured TA muscles revealed increased GFP^+^ cells in i*Ezh2*^−/−^;*PDGFRα*^*EGFP*^ mice (Fig. 2c). FAP expansion remained higher in i*Ezh2*^−/−^;*PDGFRα*^*EGFP*^ mice at 28 dpi (Fig. 2d), when mice developed fibrosis (Fig. 1f). After injury, macrophage-secreted TNF induces FAP apoptosis to limit ECM production during regeneration^14^. Thus, the increased number of FAPs in the i*Ezh2*^−/−^;*PDGFRα*^*EGFP*^ mice may be due to either decreased FAP apoptosis or increased proliferation. To distinguish between these two possibilities, we performed a TUNEL assay on muscle cross-sections from injured TA muscle of control *PDGFRα*^*EGFP*^ or i*Ezh2*^−/−^;*PDGFRα*^*EGFP*^ mice and observed no significant differences in the number of apoptotic GFP^+^/TUNEL^+^ cells (Extended Data Fig. 2b). To analyze FAP proliferation, control *PDGFRα*^*EGFP*^ and *iEzh2*^−/−^;*PDGFRα*^*EGFP*^ mice were injured with NTX and intraperitoneally injected with 5-ethynyl-2′-deoxyuridine (EdU). EdU incorporation of FAPs in muscle sections of i*Ezh2*^−/−^;*PDGFRα*^*EGFP*^ was more than 50% higher compared to control *PDGFRα*^*EGFP*^ mice (Fig. 2e). Thus, *Ezh2* deletion in adult MuSCs is associated with reduced MuSC proliferation and increased FAP proliferation.

**Fig. 3:**
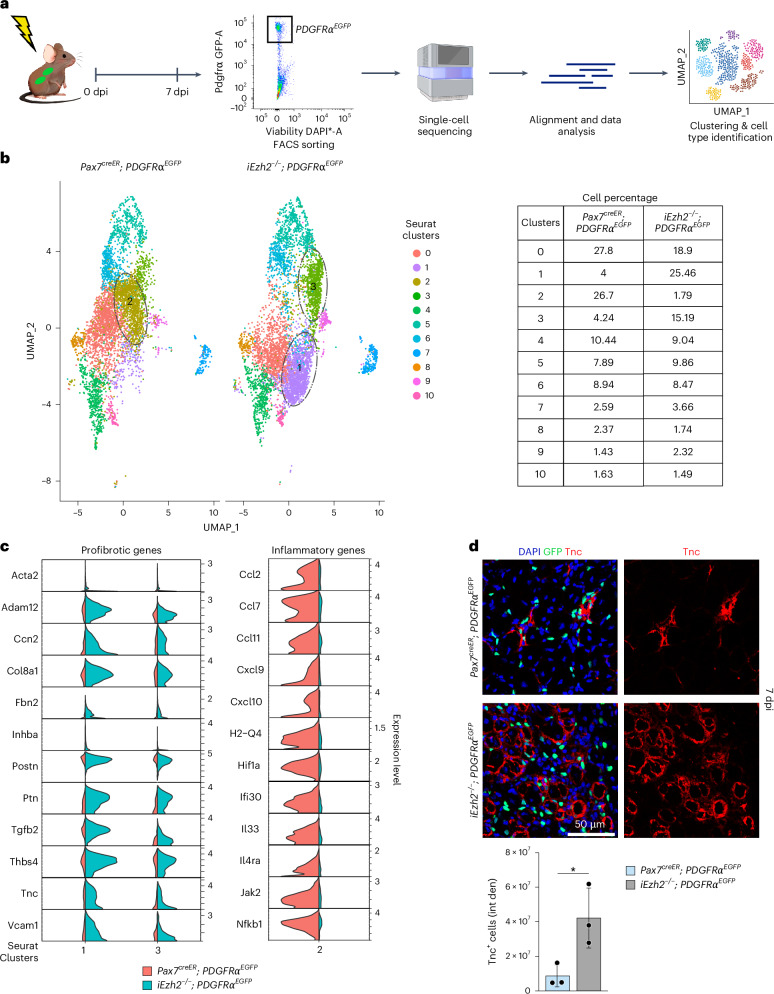
FAPs isolated from i*Ezh2*^−/−^;*PDGFRα*^*EGFP*^ mice express pro-fibrotic transcripts. **a**, Schematic of the scRNA-seq protocol. FAPs were FACS-isolated based on GFP expression. **b**, scRNA-seq UMAP plots of control *PDGFRα*^*EGFP*^ and i*Ezh2*^−/−^;*PDGFRα*^*EGFP*^ mice showing FAP clusters. Clusters significantly modulated (>1.5 fold change) in i*Ezh2*^−/−^;*PDGFRα*^*EGFP*^ compared to controls are circled. Right panel shows a table with the relative cell percentages for each cluster. Two mice were employed per single scRNA-seq experiment, and the experiment was performed twice, resulting in a total sample size of *n* = 4 mice. A total of 10,857 cells were analyzed. **c**, Violin plots of fibrotic (left) and inflammatory (right) gene expression in the indicated clusters. **d**, Tenascin immunostaining of TA muscle sections from control *PDGFRα*^*EGFP*^ or i*Ezh2*^−/−^;*PDGFRα*^*EGFP*^ mice at 7 dpi. Nuclear GFP reporter identifies FAPs; DAPI marks nuclei. Bottom panel shows quantification of Tnc^+^ cells in control *PDGFRα*^*EGFP*^ or i*Ezh2*^−/−^;*PDGFRα*^*EGFP*^ mice. Two muscle sections per mouse were analyzed. Data are presented as mean ± s.d., two-tailed unpaired *t*-test, *n* = 3 mice,^*^P = 0.0353. Source data

To gain insights and characterize the PDGFRα^EGFP^ cell population, we FACS-isolated GFP^+^ cells from TA muscles of control *PDGFRα*^*EGFP*^ or i*Ezh2*^−/−^;*PDGFRα*^*EGFP*^ mice at 7 dpi and conducted single-cell RNA sequencing (scRNA-seq) (Fig. 3a). Uniform manifold approximation and projection (UMAP) clustering partitioned GFP^+^ cells isolated from either control *PDGFRα*^*EGFP*^ or i*Ezh2*^−/−^;*PDGFRα*^*EGFP*^ mice into 10 clusters (Fig. 3b). The transcriptomes of the individual clusters are reported in Supplementary Table 2. The percentage of cells corresponding to clusters 1 and 3 was increased in i*Ezh2*^−/−^;*PDGFRα*^*EGFP*^ mice (6.5-fold for cluster 1 and 3.6-fold for cluster 3) (Fig. 3b). Clusters 1 and 3 are composed of FAPs expressing pro-fibrotic transcripts (Fig. 3c)^30–32^. Acta2 is upregulated in hepatic stellate cells as they transition into myofibroblasts during liver injury. Mice lacking *Acta2* exhibited significantly less liver fibrosis after chronic injury, indicating a critical role of this gene in fibrogenesis^33^. Vcam1 and Adam12 are expressed in pro-fibrotic FAPs of dystrophic mice (Fig. 3c)^15^. Ccn2 (CTGF) is a matricellular protein with pro-fibrotic properties overexpressed in muscle dystrophies. Reducing Ccn2 levels in *mdx* dystrophic mice decreases fibrosis and improves skeletal muscle phenotype and function^34^. Collagen type VIIIα1 chain (Col8a1) is an ECM component^35^. Transforming growth factor beta-2 (TGFβ2) is a key mediator of the fibrotic response associated with increased fibrosis in dystrophic muscle^36^. Periostin (Postn) is a secreted ECM protein functioning upstream of TGFβ. *Postn* deletion reduces muscular dystrophy and fibrosis by modulating the TGFβ pathway^37^. FAPs expressing Tenascin-C (Tnc), a protein driving persistent fibrosis^38^, were increased in clusters 1 and 3, and the Tnc protein was highly expressed in muscle sections of 7-dpi i*Ezh2*^−/−^;*PDGFRα*^*EGFP*^ mice (Fig. 3d). FAP-secreted Tnc maintains MuSC quiescence^39^, suggesting the possibility that, besides promoting fibrosis, excessive Tnc signaling may constrain MuSC activation. Cluster 2, composed of cells expressing transcripts related to inflammation and other processes, was decreased (14.8-fold) in i*Ezh2*^−/−^;*PDGFRα*^*EGFP*^ mice (Fig. 3c). Cluster 2 FAPs expressed: (1) the chemokine Ccl2, a ligand of the inflammatory-related CC-chemokine-receptor 2 (Ccr2). Ccr2 signaling is critical to mount the inflammatory response required for muscle regeneration, and *Ccl2* deletion adversely impacts muscle regeneration^40^; (2) Cxcl9, a member of the CXC chemokine family, which exerts anti-fibrotic functions by inhibiting TGFβ^41^; (3) interleukin 33 (IL-33). IL-33-expressing FAPs facilitate the establishment of the pro-regenerative niche after muscle injury and are reduced in old mice^42,43^; and (4) the alpha chain of the interleukin 4 receptor (IL-4Rα). After muscle injury, eosinophils secrete IL-4, which activates FAPs to support myogenesis and inhibit FAP adipogenesis^44^. Thus, deleting *Ezh2* in MuSCs preferentially enhances the proliferation of FAPs expressing pro-fibrotic genes while reducing a FAP population that expresses genes associated with muscle regeneration.

### i*Ezh2*^−/−^ MuSCs promote FAP proliferation

**Fig. 4:**
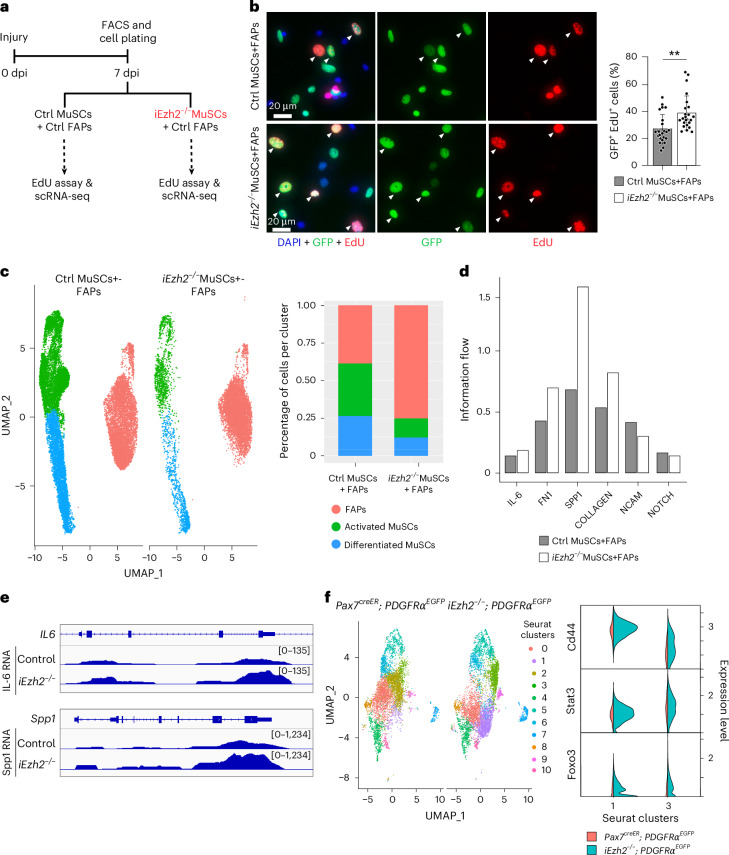
i*Ezh2*^−/−^ MuSCs promote FAP proliferation. **a**, Schematic of FACS isolation and plating of MuSCs and FAPs. After 72 hours in culture, EdU assay and scRNA-seq were performed. **b**, EdU immunostaining on co-cultured MuSCs and GFP^+^ FAPs. MuSCs were isolated from either control *PDGFRα*^*EGFP*^ (Ctrl MuSCs) or i*Ezh2*^−/−^;*PDGFRα*^*EGFP*^ (i*Ezh2*^−/−^ MuSCs) mice, whereas FAPs were isolated from control *PDGFRα*^*EGFP*^ mice. Arrows indicate GFP^+^EdU^+^ cells. Right panel shows quantification of *PDGFRα*^*EGFP*+^EdU^+^ cells. Data are presented as mean ± s.d., two-tailed unpaired *t*-test, *n* = number of regions counted (*n* = 23), with cells pooled from three independent experiments ^**^P = 0.0015. **c**, scRNA-seq UMAP plots of co-cultured MuSCs and FAPs isolated from control *PDGFRα*^*EGFP*^ or i*Ezh2*^−/−^;*PDGFRα*^*EGFP*^ mice. Bar plots on the right show the relative percentage of cell clusters. **d**, Information flow showing context-specific signaling pathways enriched in co-culture conditions, with signaling directed from MuSCs to FAPs. **e**, RNA-seq tracks of IL-6 and Spp1 transcripts in control or i*Ezh2*^−/−^ MuSCs at 7 dpi and 3 dpi, respectively. **f**, scRNA-seq UMAP (from Fig. 3b) and violin plots showing transcripts expressed in the indicated clusters in control *PDGFRα*^*EGFP*^ or i*Ezh2*^−/−^;*PDGFRα*^*EGFP*^ mice. Source data

**Extended Data Fig. 3:**
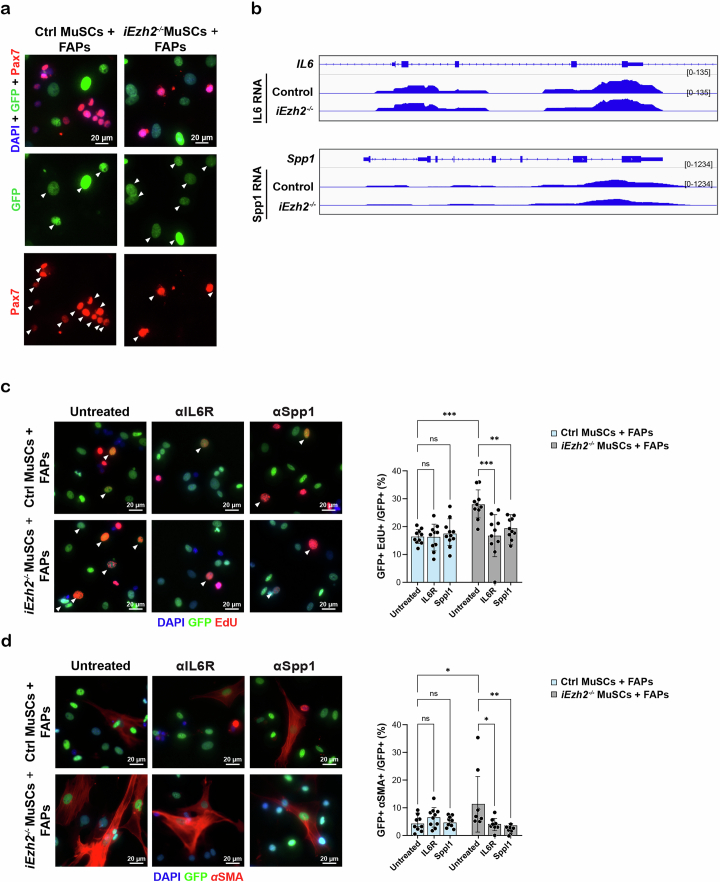
Reduced number of Pax7^+^ cells in i*Ezh2*^−/−^ MuSCs and FAPs co-cultures; IL-6 and Spp1 expression in FAPs from control or i*Ezh2*^−/−^ mice. **a**, Representative images of co-cultures of control or i*Ezh2*^−/−^ MuSCs and FAPs. The number of Pax7^+^ cells is reduced and FAPs are increased in i*Ezh2*^−/−^ MuSCs and FAPs co-cultures. The experiment was independently repeated three times with similar results. **b**, Bulk-RNA-seq tracks of IL-6 and Spp1 in FAPs isolated from either control or i*Ezh2*^−/−^ mice. **c**, EdU incorporation in co-cultures of MuSCs and *PDGFRα*^*EGFP*+^ FAPs untreated or treated with either IL-6R or Spp1 neutralizing antibodies. MuSCs were isolated from either control *PDGFRα*^*EGFP*^ or i*Ezh2*^−/−^;*PDGFRα*^*EGFP*^ mice; FAPs were isolated from control *PDGFRα*^*EGFP*^ mice. Arrows indicate GFP^+^EdU^+^ cells. Right panel shows quantification of *PDGFRα*^*EGFP*+^EdU^+^ cells. n= number of regions counted (n = 10), with cells pooled from three independent experiments. ^**^P = 0.0069, ^***^P = 0.0004 (untreated Ctrl MuSCs vs. untreated *i*Ezh2^−/−^ MuSCs), ^***^P = 0.0005 (untreated *i*Ezh2^−/−^ MuSCs vs. IL6R-treated *i*Ezh2^−/−^ MuSCs), ns= non-significant. **d**, αSMA immunostaining of co-cultured MuSCs and *PDGFRα*^*EGFP*+^ FAPs untreated or treated with either IL-6R antibody or Spp1 neutralizing antibodies. MuSCs were isolated as described above. Right panel shows quantification of *PDGFRα*^*EGFP*+^αSMA^+^ cells. n= number of regions counted (n = 10), with cells pooled from three independent experiments. ^*^P = 0.0223 (untreated Ctrl MuSCs vs. untreated *i*Ezh2^−/−^ MuSCs), ^*^P = 0.0169 (untreated *i*Ezh2^−/−^ MuSCs vs. IL6R-treated *i*Ezh2^−/−^ MuSCs), ^**^P = 0.0038, ns= non-significant. Two-way ANOVA with Sidak’s multiple comparisons (**c, d**). Source data

Muscle homeostasis and regeneration are regulated by MuSCs, FAPs and infiltrating immune cell types^45–48^. Having observed an increased number of FAPs in i*Ezh2*^−/−^ muscles, we sought to determine whether MuSCs from i*Ezh2*^−/−^ mice could independently drive FAP proliferation, without the influence of other cell types. Control or i*Ezh2*^−/−^ MuSCs FACS-isolated from 7-dpi mice were co-cultured with *PDGFRα*^*EGFP*^ FAPs isolated from 7-dpi control mice. After 72 hours, we assessed EdU incorporation to measure entry into the S phase of the cell cycle and carried out scRNA-seq (Fig. 4a). i*Ezh2*^−/−^ co-cultures with FAPs showed a higher percentage of *PDGFRα*^*EGFP*+^EdU^+^ cells, compared to controls, indicating that i*Ezh2*^−/−^ MuSCs are sufficient to stimulate FAP proliferation (Fig. 4b). scRNA-seq analysis revealed clusters corresponding to activated MuSCs, differentiating primary myocytes and FAPs (Fig. 4c). Consistent with decreased MuSC proliferation in i*Ezh2*^−/−^ mice (Fig. 1d), i*Ezh2*^−/−^ co-cultures contained fewer MuSCs (Fig. 4c and Extended Data Fig. 3a). However, the number of FAPs in i*Ezh2*^−/−^ MuSC co-cultures was higher compared to control co-cultures (Fig. 4c and Extended Data Fig. 3a). Next, we analyzed scRNA-seq data to explore ligand−receptor interactions between MuSCs and FAPs. Cell−cell communication analysis^49^ predicted enriched collagen, fibronectin-1 (FN1), IL-6 and Spp1 signaling directed from i*Ezh2*^−/−^ MuSCs to FAPs (Fig. 4d). Confirming signal directionally, bulk RNA-seq displayed increased expression of IL-6 and Spp1 in i*Ezh2*^−/−^ MuSCs, not in FAPs of i*Ezh2*^−/−^ mice (Fig. 4e and Extended Data Fig. 3b). FAP clusters 1 and 3 were enriched for cells expressing the Spp1 receptor Cd44 (ref. ^50^) and transcription factors Stat3 and FoxO3, which mediate IL-6 signaling (Fig. 4f). To assess the functional relevance of IL-6 and Spp1 signaling in inducing FAP expansion, we co-cultured 7-dpi control *PDGFRα*^*EGFP*^ FAPs with 7-dpi MuSCs derived from either control or i*Ezh2*^−/−^ mice for 48 hours in the absence or presence of IL-6 receptor (IL-6R) or Spp1 neutralizing antibodies. EdU incorporation assay confirmed increased proliferation of FAPs co-cultured with i*Ezh2*^−/−^ MuSCs compared to control MuSCs (Extended Data Fig. 3c). Blockade with either IL-6R or Spp1 neutralizing antibodies resulted in decreased FAP proliferation (Extended Data Fig. 3c). scRNA-seq on FAPs FACS-isolated from control *PDGFRα*^*EGFP*^ or i*Ezh2*^−/−^;*PDGFRα*^*EGFP*^ mice at 7 dpi showed increased expression of pro-fibrotic transcripts in FAPs derived from mutant mice (Fig. 3c). In agreement with these data, co-cultures with i*Ezh2*^−/−^ MuSCs contained a higher percentage of fibrogenic αSMA^+^ FAPs, which decreased upon IL-6 or Spp1 blockade (Extended Data Fig. 3d). These findings indicate that (1) i*Ezh2*^−/−^ MuSCs alone can enhance FAP proliferation; (2) i*Ezh2*^−/−^ MuSCs express higher levels of IL-6 and Spp1 compared to control MuSCs; (3) FAPs isolated from i*Ezh2*^−/−^ muscles are enriched for the Spp1 receptor Cd44 and IL-6 signaling components Stat3 and FoxO3; and (4) IL-6 and Spp1 secreted from i*Ezh2*^−/−^ MuSCs are required to induce FAP proliferation and fibrogenic differentiation.

### Aging muscles display regenerative defects, fibrosis and an increased number of FAPs

**Fig. 5:**
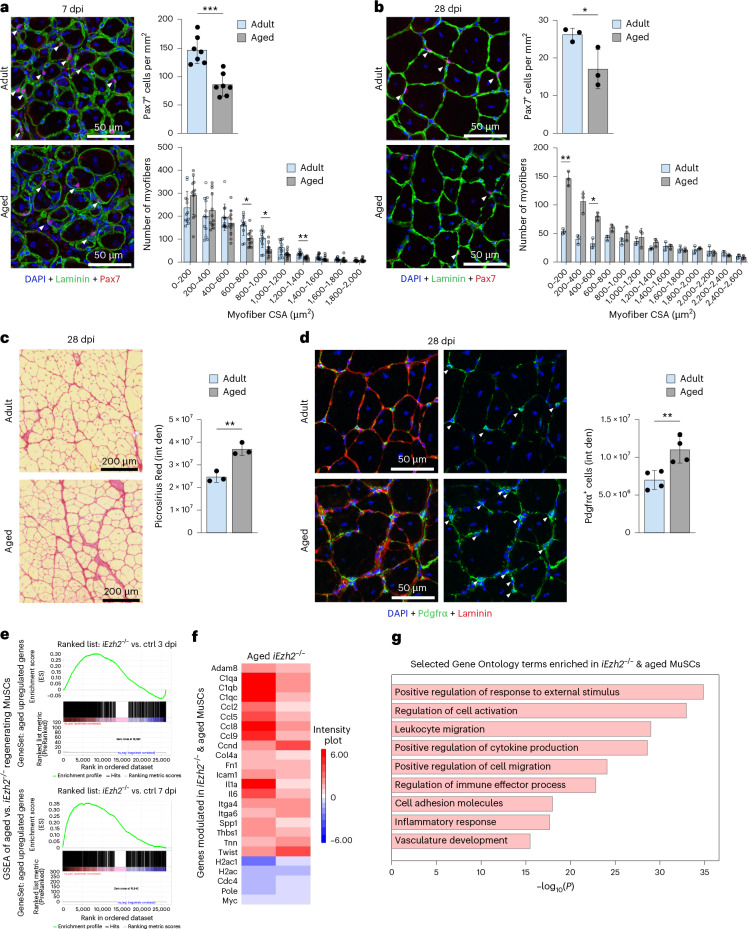
Aging muscles display regenerative defects, worsened fibrosis and increased number of FAPs. **a**,**b**, Laminin and Pax7 immunostaining of TA muscle sections from adult (3-month-old) or aged (24-month-old) mice at 7 dpi (**a**) and 28 dpi (**b**). DAPI marks nuclei. Quantification of Pax7^+^ cells and myofiber CSA in adult or aged mice is shown for both timepoints. At least two muscle sections per mouse were analyzed. Data are presented as mean ± s.d., two-tailed unpaired *t*-test. **a**, *n* = 7 mice for Pax7 quantification, ^***^P = 0.0002; *n* = 12 from seven adult or aged mice, respectively, for CSA analysis, ^*^P = 0.0151 (600−800-μm^2^ fibers), ^*^P = 0.0142 (800–1,000-μm^2^ fibers), ^**^P = 0.0011 (1,200−1,400-μm^2^ fibers). **b**, *n* = 3 mice for Pax7 quantification, ^*^P = 0.044; *n* = 3 for CSA analysis, ^*^P = 0.0250 (400−600-μm^2^ fibers), ^**^P = 0.0042 (0−200-μm^2^ fibers). **c**, Picrosirius Red staining and quantification of TA muscle sections from adult and aged mice at 28 dpi. Two muscle sections per mouse were analyzed. Data are presented as mean ± s.d., two-tailed unpaired *t*-test, *n* = 3 mice, ^**^P = 0.004. **d**, Pdgfrα and Laminin immunostaining of TA muscle sections from adult and aged mice at 28 dpi. DAPI marks nuclei. Quantification of Pdgfrα^+^ cells is shown on the right. Two muscle sections per mouse were analyzed. Data are presented as mean ± s.d., two-tailed unpaired *t*-test, *n* = 4 mice, ^**^P = 0.0096. **e**, GSEA plots comparing gene expression of aged and i*Ezh2*^−/−^ regenerating MuSCs. The *y* axis represents the ranking metric score; the *x* axis shows the ranked list of genes. **f**, Heat map of selected genes differentially expressed in i*Ezh2*^−/−^ and aged MuSCs. Gene names are shown on the *y* axis. **g**, Bar plot of selected Gene Ontology terms enriched in i*Ezh2*^−/−^ and aged MuSCs. Source data

*iEzh2*^−/−^ mice exhibit reduced MuSC number and proliferation, impaired regeneration as well as increased fibrosis. These phenomena were also observed in aged mice. Defective regeneration was evident from the decreased number of MuSCs and reduced CSA in aged (approximately 24-month-old) mice compared to adult (approximately 3-month-old) mice, at both 7 dpi and 28 dpi (Fig. 5a,b). Additionally, collagen deposition and FAPs (Pdgfrα^+^) were elevated in aged mice compared to adult mice at 28 dpi (Fig. 5c,d). Transcriptomics analysis of i*Ezh2*^−/−^ MuSCs at 3 dpi and 7 dpi and aged MuSCs at 3 dpi^23^ revealed a significant positive correlation between transcripts upregulated in i*Ezh2*^−/−^ and aged MuSCs, including IL-6 and Spp1 (Fig. 5e−g and Supplementary Table 3).

### Aged MuSCs promote FAP proliferation through IL-6 and Spp1

**Fig. 6:**
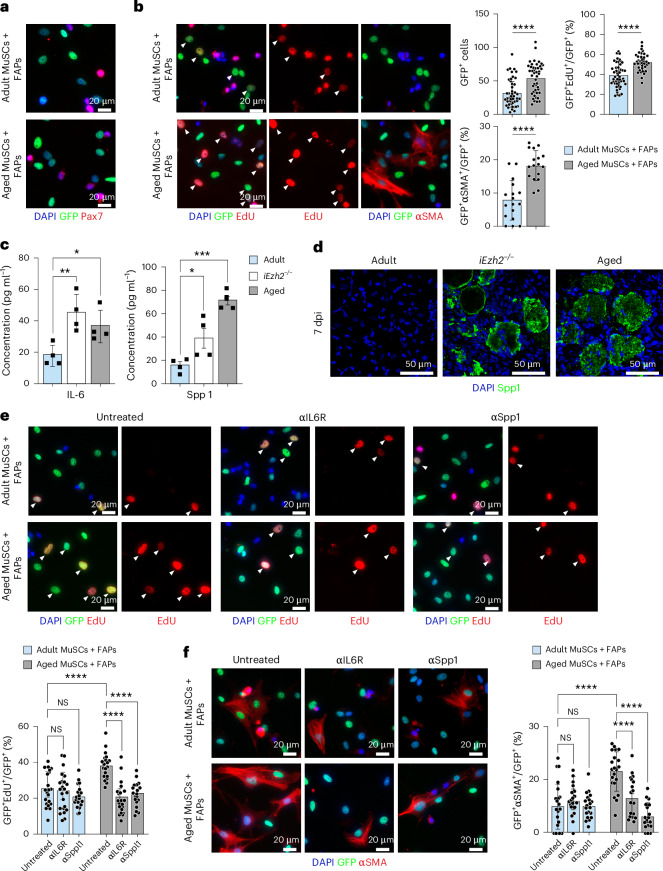
Aged MuSCs promote FAP proliferation through the secretion of IL-6 and Spp1. **a**, Representative images of Pax7 immunostaining and GFP reactivity of co-cultures with MuSCs isolated from either adult (top panel) or aged (bottom panel) (24-month-old) mice at 3 dpi and FAPs isolated from *Pax7*^*creER*^;*PDGFRα*^*EGFP*^ adult mice at 3 dpi (*n* = 3 independent experiments). **b**, EdU incorporation, GFP reactivity and αSMA immunostaining of co-cultured MuSCs and GFP^+^ FAPs. Arrows indicate *PDGFRα*^*EGFP*+^EdU^+^ cells. Right panel shows quantification of *PDGFRα*^*EGFP*+^, *PDGFRα*^*EGFP*+^EdU^+^ and *PDGFRα*^*EGFP*+^αSMA^+^ cells. *n* = number of regions counted, with cells pooled from three independent experiments (*n* = 45 for *PDGFRα*^*EGFP*+^ and *PDGFRα*^*EGFP*+^EdU^+^ and *n* = 17 for *PDGFRα*^*EGFP*+^αSMA^+^), ^****^P < 0.0001, two-tailed unpaired *t*-test. **c**, Bar plots showing IL-6 and Spp1 concentrations in the supernatants from adult, i*Ezh2*^−/−^ or aged MuSCs. Data are presented as mean ± s.d. (*n* = 4 independent experiments) ^*^P = 0.0461 (IL-6 panel) ^*^P = 0.0321 (Spp1 panel), ^**^P = 0.0069 (IL-6 panel), ^***^P = 0.0001 (Spp1 panel). One-way ANOVA with Dunnett’s multiple comparison. **d**, Spp1 immunostaining of TA muscle sections from adult, i*Ezh2*^−/−^ and aged mice at 7 dpi. DAPI marks nuclei. Three muscle sections per mouse were analyzed (*n* = 3 mice). **e**, EdU incorporation in co-cultures of MuSCs and *PDGFRα*^*EGFP*+^ FAPs untreated or treated with either IL-6R or Spp1 neutralizing antibodies. MuSCs were isolated from either adult or aged mice; FAPs were isolated from *Pax7*^*creER*^;*PDGFRα*^*EGFP*^ mice. Arrows indicate GFP^+^EdU^+^ cells. Bottom panel shows quantification of *PDGFRα*^*EGFP*+^EdU^+^ cells. *n* = number of regions counted, with cells pooled from three independent experiments (*n* = 22) ^****^P = < 0.0001. **f**, αSMA immunostaining of co-cultured MuSCs and *PDGFRα*^*EGFP*+^ FAPs untreated or treated with either IL-6R antibody or Spp1 neutralizing antibodies. MuSCs were isolated as described above. Right panel shows quantification of *PDGFRα*^*EGFP*+^αSMA^+^ cells. *n* = number of regions counted, with cells pooled from three independent experiments (*n* = 22) ^****^P = < 0.0001, NS, non-significant. Two-way ANOVA with Sidak’s multiple comparisons (**e**,**f**). αIL6R, against-IL6R antibody; αSppl1, against-Spp1 antibody. Source data

The increased fibrosis and FAP number observed in both i*Ezh2*^−/−^ and aged mice, coupled with the ability of i*Ezh2*^−/−^ MuSCs to promote FAP proliferation, led us to examine the behavior of aged MuSCs when co-cultured with FAPs. MuSCs FACS-isolated from either adult (approximately 3-month-old) or aged (approximately 24-month-old) mice at 3 dpi were co-cultured with FAPs FACS-isolated from adult *PDGFRα*^*EGFP*^ mice at 3 dpi (Fig. 6a). After 48 hours, we assessed FAP proliferation through EdU incorporation of GFP^+^ cells. Co-cultures of aged MuSCs showed an increased number of FAPs and a higher percentage of GFP^+^EdU^+^ cells compared to those with adult MuSCs (Fig. 6b). Additionally, a greater proportion of FAPs co-cultured with aged MuSCs expressed α-smooth muscle actin (αSMA), a marker of FAP-derived pro-fibrotic myofibroblasts^13,51^ (Fig. 6b). We also evaluated the presence of IL-6 and Spp1 proteins in the supernatants of FACS-isolated and cultured adult, i*Ezh2*^−/−^ and aged MuSCs. Both IL-6 and Spp1 were enriched in the supernatants of i*Ezh2*^−/−^ and aged MuSCs (Fig. 6c). Furthermore, Spp1 was detected in muscle sections of i*Ezh2*^−/−^ and aged mice (Fig. 6d). To assess the functional relevance of IL-6 and Spp1 signaling, we co-cultured FACS-isolated MuSCs from adult or aged mice at 3 dpi with adult FAPs for 48 hours in the absence or presence of IL-6R or Spp1 neutralizing antibodies and assessed FAP proliferation by EdU incorporation. The number of FAPs was higher in co-cultures with aged compared to adult MuSCs. Blockade of either IL-6 or Spp1 resulted in reduced FAP proliferation in aged but not adult MuSC-FAP co-cultures (Fig. 6e). Moreover, co-cultures with aged MuSCs contained a higher percentage of fibrogenic αSMA^+^ FAPs, which decreased upon IL-6 or Spp1 blockade (Fig. 6f). The percentage of αSMA^+^ FAPs in adult MuSC co-cultures was not affected by IL-6 or Spp1 blockade.

### H3K27me3 erosion at the *Nfkb1* gene is associated with increased NF-κB expression and activation of IL-6 and Spp1 in aged MuSCs

**Fig. 7:**
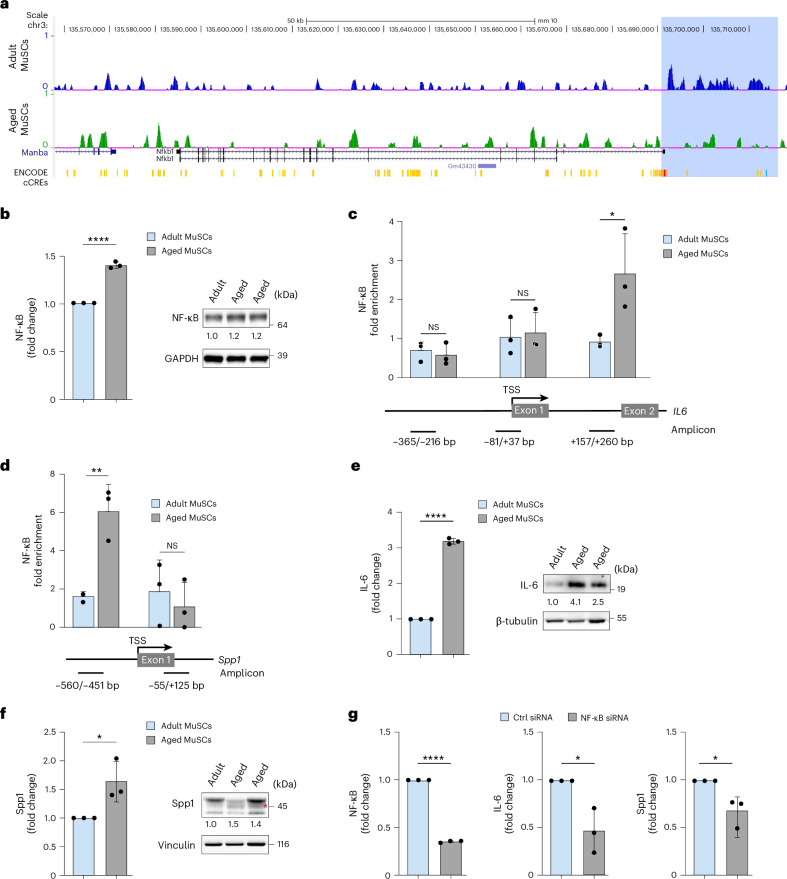
Reduced H3K27me3 is associated with increased NF-κB expression and IL-6 and Spp1 activation in aged MuSCs. **a**, Representative UCSC browser tracks of H3K27me3 CUT&RUN analysis at the *Nfkb1* locus in adult (3-month-old) and aged (24-month-old) MuSCs at 3 dpi. The H3K27me3 signal was normalized against control IgG. ENCODE cCREs indicate predicted promoter (red), proximal (orange) and distal enhancers (yellow) and CTCF binding site (blue). The shaded area highlights predicted *Nfkb1* promoter and enhancer elements with reduced H3K27me3 signal in aged MuSCs. **b**, Quantification of NF-κB mRNA and protein levels in adult and aged MuSCs at 3 dpi. In the immunoblot, numbers below the images indicate the fold enrichment of NF-κB normalized to GAPDH. Quantification was determined with ImageJ software. NF-κB mRNA expression is presented as mean ± s.d.; *n* = 3 independent experiments. ^****^P < 0.0001, two-tailed unpaired *t*-test. **c**,**d**, CUT&RUN-qPCR assay at the *IL6* and *Spp1* using chromatin from adult or aged MuSCs at 3 dpi. NF-κB antibody was used, with IgG as a negative control. Amplified regions are indicated relative to the TSS. Data are presented as mean ± s.d.; *n* = 3 independent experiments. Fold enrichment = 2^(ΔCt(Target) − ΔCt(IgG))^, ^*^P = 0.046, ^**^P = 0.005, NS, non-significant, two-tailed unpaired *t*-test. **e**, IL-6 mRNA and protein levels of adult and aged MuSCs at 3 dpi. In the immunoblot, numbers below the images indicate fold enrichment of IL-6 normalized to β-tubulin. Quantification was performed using ImageJ software. IL-6 mRNA expression is presented as mean ± s.d.; *n* = 3 independent experiments. ^****^P = < 0.0001, two-tailed unpaired *t*-test. **f**, Spp1 mRNA and protein levels in adult and aged MuSCs at 3 dpi. In the immunoblot, numbers below the images indicate fold enrichment of Spp1 normalized to vinculin. Quantification was performed using ImageJ software. Spp1 mRNA expression is presented as mean ± s.d.; *n* = 3 independent experiments. ^*^P = 0.0368, two-tailed unpaired *t*-test. **g**, NF-κB, IL-6 and Spp1 expression in aged MuSCs transfected with control or NF-κB siRNA. Data are presented as mean ± s.d.; *n* = 3 independent experiments, ^*^P = 0.017 (IL-6 panel), ^*^P = 0.025 (Spp1 panel), ^****^P < 0.0001 (NF-κB panel), two-tailed unpaired *t*-test. cCREs, candidate *cis*-regulatory elements; chr3, chomosome 3. Source data

**Extended Data Fig. 4:**
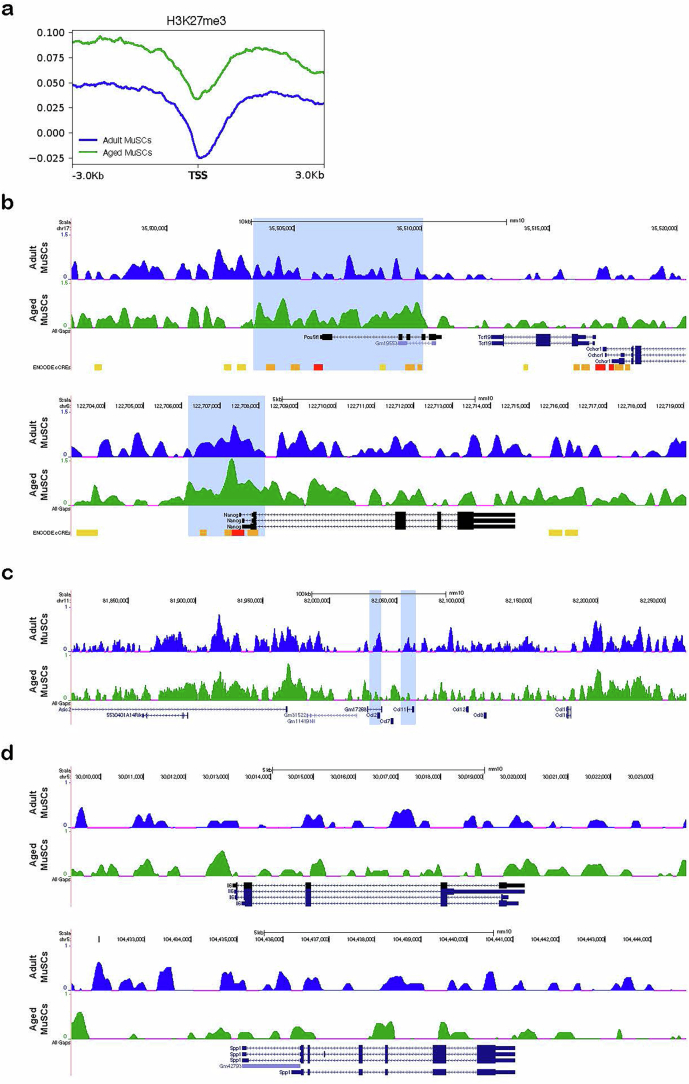
Global and gene-specific H3K27me3 enrichment in adult and aged MuSCs. **a**,Metaplot of H3K27me3 signal centered at the transcriptional start site (TSS) and extending -/+3Kb in adult and aged (24-month-old) MuSCs. **b**,Representative UCSC browser tracks of H3K27me3 CUT&RUN assay at the pluripotency genes *Pou5f1 (Oct4)* and *Nanog* in adult and aged MuSCs. The shaded area highlights predicted promoter and enhancer elements with increased H3K27me3 signal in aged MuSCs. **c**, Representative UCSC browser tracks of H3K27me3 CUT&RUN assay at the aging-associated cytokines *Ccl2* and *Ccl11* genes in adult and aged MuSCs. The shaded area highlights *Ccl2* and *Ccl11* gene regions with decreased H3K27me3 signal in aged MuSCs **d**, Representative UCSC browser tracks of H3K27me3 CUT&RUN assay at the *IL6* and *Spp1* genes in adult and aged MuSCs. The H3K27me3 signals in all panels were normalized to IgG controls. ENCODE cCRE annotations indicate predicted promoter (red), proximal enhancer (orange), and distal enhancer (yellow) elements. Source data

To investigate the mechanisms underlying the increased IL-6 and Spp1 expression in aged MuSCs, we compared the genome-wide distribution of the repressive epigenetic mark H3K27me3 in adult and aged MuSCs using a CUT&RUN assay. Globally, H3K27me3 levels were elevated in aged MuSCs (Extended Data Fig. 4a). H3K27me3 was increased at key pluripotency regulators *Oct4* (*Pou5f1*) and *Nanog* (Extended Data Fig. 4b). These genes are among the 30% of genes that are transcriptionally repressed in both adult and aged MuSCs and acquire further H3K27me3 in aged MuSCs^52^. The pro-inflammatory cytokines Ccl2 and Ccl11 are highly expressed in aged MuSCs and in the plasma of aging mice^23,53,54^. Correlating with their increased expression, H3K27me3 was decreased at both *Ccl2* and *Ccl11* loci in aged MuSCs (Extended Data Fig. 4c). By contrast, although IL-6 and Spp1 levels were increased in aged MuSCs, H3K27me3 enrichment at these genes did not differ between adult and aged MuSCs (Extended Data Fig. 4d). Aged MuSCs and their niche exhibit elevated levels of pro-inflammatory cytokines and matrix remodeling factors^23,55,56^, many of which are regulated by NF-κB signaling. NF-κB expression is increased in both murine and human aged MuSCs^23,54^. Consistent with the reported increased NF-κB expression, in aged MuSCs H3K27me3 was reduced at the *Nfkb1* promoter and predicted enhancer regions (Fig. 7a), correlating with increased chromatin accessibility at this locus^57^. These epigenetic changes were accompanied by increased NF-κB mRNA and protein levels in aged MuSCs (Fig. 7b). Furthermore, increased NF-κB expression in aged MuSCs was associated with enhanced NF-κB binding immediately downstream of the transcriptional start site (TSS) of the *IL6* gene and at the promoter region of the *Spp1* gene (Fig. 7c,d). Accordingly, IL-6 and Spp1 mRNA and protein levels were higher in aged compared to adult MuSCs (Fig. 7e,f). To assess the functional relevance of NF-κB in driving IL-6 and Spp1 expression, we performed small interfering RNA (siRNA)-mediated knockdown of NF-κB in aged MuSCs. Compared to control siRNA, NF-κB knockdown led to reduced expression of both IL-6 and Spp1 transcripts (Fig. 7g). Taken together, these results suggest that reduced H3K27me3 at the *Nfkb1* promoter in aged MuSCs is associated with increased NF-κB expression, enhanced recruitment to target genes and subsequent activation of Spp1 and IL-6 transcription.

### IL-6 and Spp1 blockade reduces fibrosis in aged injured mice

**Fig. 8:**
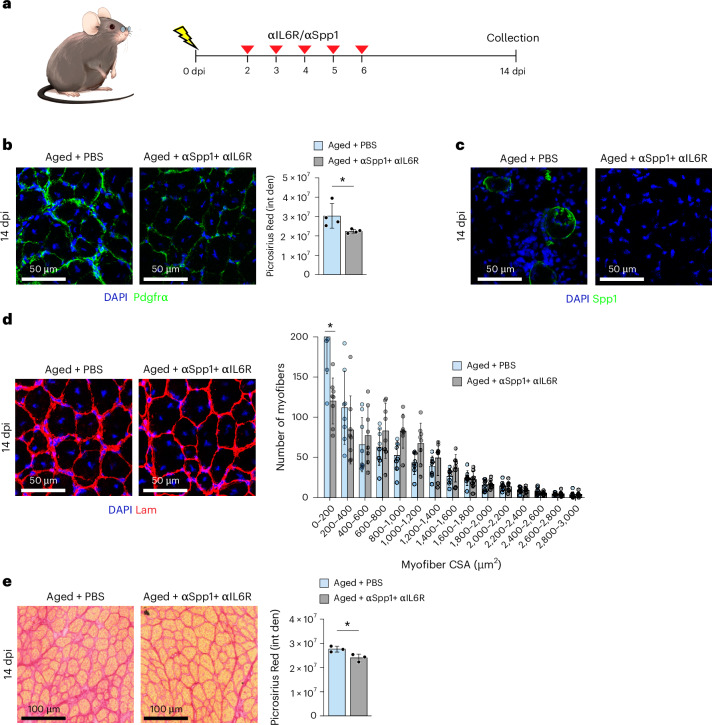
IL-6 and Spp1 blockade reduces fibrosis in aged injured mice. **a**, Schematic of IL-6R and Spp1 antibodies injection in aged (24-month-old) mice. **b**, Pdgfrα immunostaining of TA muscle sections from adult or aged mice injected with either PBS or Spp1 and IL-6R antibodies at 14 dpi. DAPI identifies cell nuclei. On the right is the quantification of Pdgfrα^+^ cells. Two muscle sections per mouse were analyzed. Data are presented as mean ± s.d., two-tailed unpaired *t*-test, *n* = 4 mice, ^*^P = 0.0425. **c**, Representative images of Spp1 immunostaining of TA muscle sections from aged mice injected with either PBS or Spp1 and IL-6R antibodies at 14 dpi. DAPI identifies cell nuclei. The experiment was independently repeated three times with similar results. **d**, Laminin immunostaining of TA muscle sections from aged mice treated with PBS (untreated) or with Spp1 and IL-6R antibodies at 14 dpi. DAPI identifies cell nuclei. Right panel shows quantification of myofiber CSA analyzed. Two muscle sections per mouse were analyzed. Data are presented as mean ± s.d., two-tailed unpaired *t*-test. *n* = 8 TAs from four PBS-treated or against-Spp1 antibody-treated and against-IL6R antibody-treated mice, respectively, ^*^P = 0.0197 (0−200-μm^2^ fibers). **e**, Picrosirius Red staining and quantification of fibrotic area in TA muscle sections from aged mice injected with PBS or with Spp1 and IL-6R antibodies at 14 dpi. Two muscle sections per mouse were analyzed. Data are presented as mean ± s.d., two-tailed unpaired *t*-test. *n* = 6 TA from three PBS-treated or against-Spp1 antibody-treated and against-IL-6R antibody-treated mice-treated mice, respectively, ^*^P = 0.0338. αIL6R, against-IL6R antibody; αSppl1, against-Spp1 antibody. Source data

To assess the translational relevance of our co-culture findings to in vivo conditions, we treated aged (24-month-old) mice with IL-6R and Spp1 neutralizing antibodies. Mice were injured with NTX and, 48 hours later, received intramuscular injections with either PBS or IL-6R+Spp1 neutralizing antibodies for five consecutive days. Muscles were collected at 14 dpi (Fig. 8a). Treatment with IL-6R and Spp1 neutralizing antibodies resulted in a reduction of FAPs and undetectable levels of Spp1 (Fig. 8b,c). Additionally, IL-6 and Spp1 blockade increased CSA and reduced muscle fibrosis (Fig. 8d,e).

## Discussion

Similar to inflammaging—the chronic, low-grade inflammation associated with aging^58^—fibroaging, which refers to the tendency to develop tissue fibrosis in aging^59^, worsens regenerative capabilities and impairs skeletal muscle performance^60^. Mesenchymal and immune cells play complex roles in muscle homeostasis and regeneration, impacting physiological and pathological states and aging^45,47^. During early muscle regeneration, FAPs proliferate, supporting MuSC expansion by secreting growth factors such as IGF-1, Wnts and IL-6 (ref. ^13^). However, elevated IL-6 levels can inhibit MuSC expansion through JAK/STAT3-mediated signaling^61,62^. For instance, muscle denervation induces abnormal activation of IL-6/JAK/STAT3 signaling, leading to muscle atrophy and fibrosis, which are both mitigated by IL-6 blockade^10^. Spp1 is a pro-fibrotic secreted glycoprotein involved in several biological processes, including ECM deposition, collagen fibrillogenesis and fibrosis^63^. In dystrophic muscle, subpopulations of macrophages and T cells expressing Spp1 are chronically activated^64,65^, and Spp1 levels are elevated in aging macrophages and MuSCs^23,66,67^. Deletion of *Spp1* improves muscle dystrophy by promoting a pro-regenerative macrophage phenotype^68^ and enhances insulin sensitivity in mice lacking the glucose transporter *Glut1/Slc2a1* in endothelial cells. Spp1 has a distinct regulation in aging macrophages, MuSCs and myofibers. Although Spp1 is increased in both aged macrophages and MuSCs, single muscle fibers of aged mice, freed of MuSCs, exhibit low levels of Spp1, which are upregulated by exercise^56^. After muscle injury, IL-6 is produced by MuSCs, macrophages, neutrophils and FAPs, promoting MuSC proliferation, fusion and differentiation^69^. However, dysregulated IL-6 expression can trigger fibroblast-to-mesenchymal transition, converting fibroblasts into αSMA^+^ myofibroblasts, leading to excessive ECM deposition and fibrosis. In this context, IL-6 also promotes expression of the pro-fibrotic Spp1 protein^70^. Aged MuSCs are prone to fibrotic conversion^8^, and fibrogenic αSMA^+^ FAPs are increased in aged mice. These FAPs secrete less matricellular WISP1, impairing MuSC expansion and commitment^71^. Furthermore, the number of PDGFRa^+^ cells expressing pro-fibrotic transcripts are increased in older adults (aged 74−99 years)^72^.

Our findings indicate that aged MuSCs play a direct role in promoting proliferation of fibrogenic FAPs through dysregulated expression and secretion of IL-6 and Spp1. Co-culture experiments revealed that aged MuSCs enhance the proliferation of fibrogenic FAPs via IL-6 and Spp1 secretion, and blocking IL-6 and Spp1 signaling in aged mice reduced the number of FAPs and decreased fibrosis. Because IL-6 and Spp1 are also secreted by macrophages and FAPs^66^, their blockade may inhibit signaling from these cell types, thereby contributing to reduced fibrosis in aged mice treated with IL-6R and Spp1 blocking antibodies. We employed IL-6R antibodies, which have been shown to efficiently reduce IL-6 levels in animal studies^73^. Moreover, the reduced Spp1 levels observed in aged mice treated with Spp1 blocking antibodies are likely due to impaired Spp1−CD44-stimulated PI3K/Akt activation, which, in turn, leads to decreased Spp1 expression^74^. The i*Ezh2*^−/−^ mouse model that we employed proved to be a valuable tool that recapitulates key features of muscle aging, such as impaired muscle regeneration and increased fibrosis. This model enabled the identification of two MuSC-derived factors—IL-6 and Spp1—that promoted FAP proliferation. Notably, both IL-6 and Spp1 were also upregulated in aged MuSCs and contributed to FAP expansion. In i*Ezh2* ^−/−^ MuSCs, H3K27me3 levels were experimentally reduced through genetic deletion of *Ezh2* (Fig. 1c). However, in aged MuSCs, reduced H3K27me3 at the *Nfkb1* promoter is not attributable to a global decline in Ezh2 expression^52^. H3K27me3 distribution and enrichement differ in adult and aged MuSCs. In aged MuSCs, transcription of approximately 30% of genes that acquire H3K27me3 remains unchanged^52^. Moreover, H3K27 acetylation is elevated at pro-fibrotic genes in aged MuSCs^75^, and enhancers are H3K27 hyperacetylated in senescent cells^76^, indicating that these regions are shielded from H3K27me3 deposition. Factors such as genome compartmentalization, condensate dynamics, chromatin accessibility, RNA-mediated enzymatic inactivation of Ezh2 and H3K27 demethylase recruitment at selected loci may mediate region-specific responses to an otherwise uniform epigenetic signal. Epigenetic erosion, characterized by the loss of fidelity and robustness of epigenetic patterns during aging, leads to dysregulated gene expression (transcriptional drift) and altered splicing^77,78^. Linking transcriptional drift, epigenetics and aging, it has been shown that RNA polymerase II elongation speed increases with age^78^ and that H3K27me3 demethylation promotes transcriptional elongation^79^. Epigenetic drift of H3K27me3 has been suggested to contribute to aging^80^, and DNA methylation becomes more heterogenous at H3K27me3 regions in aged MuSCs^53^. Suppressing transcriptional drift or reducing RNA polymerase speed extends lifespan in worms^78^. We observed that, in aged MuSCs, the H3K27me3 repressive mark was reduced at the *Nfkb1* gene, correlating with increased NF-κB levels. This increased NF-κB expression was accompanied by enhaced NF-κB recruitment at both the *IL6* and *Spp1* genes. Furthermore, NF-κB was required for *IL6* and *Spp1* activation. Our findings reveal how epigenetic changes in MuSCs drive signaling pathways that promote fibrosis with aging, offering insights into potential therapeutic strategies for age-related muscle fibrosis. Translational validation of our findings in future studies will necessitate testing larger cohorts of animals.

## Methods

All animal procedures and experimental protocols were conducted in compliance with all relevant ethical regulations and approved by National Institutes of Health (NIH), National Institute of Arthritis and Musculoskeletal and Skin Diseases (NIAMS) Animal Animal Care and Use Committee (Animal Study Proposal A023-09-04). Biosafety protocols were reviewed and approved by NIH Division of Occupational Health and Safety.

### Animal studies

Tamoxifen-inducible *Pax7*^creER^;*Ezh2*^fl/fl^ (i*Ezh2*^−/−^ mice) were generated by crossing *Pax7*^creER^ mice (B6.Cg-*Pax7*^*tm1(cre/ERT2)Gaka*/^J, The Jackson Laboratory (JAX): 017763)^21^ with *Ezh2*^*fl/fl*^ mice^22^. Control mice in ablation experiments were littermates that did not carry the Ezh2^*fl/fl*^ alleles (TmPax7-Cre^creER^;Ezh2^+/+^). i*Ezh2*^−/−^;*PDGFRα*^*EGF*P^ were generated by crossing i*Ezh2*^−/−^ mice with *PDGFRα*^*EGFP*^ mice (B6.129S4-*Pdgfra*^*tm11(EGFP)Sor*^/J, JAX: 007669).

For comparative studies, age and sex were matched in each setting. C57BL/6J (JAX: 000664) mice were from JAX. Genotyping was performed by a TaqMan-based approach provided by Transnetyx (http://www.transnetyx.com). Mice were housed in a pathogen-free facility, and all experiments were performed according to NIH Animal Care and Use regulations. Housing conditions were as follows: temperature approximately 72 °F (approximately 22 °C), 40−60% humidity and 14/10-hour light/dark daily cycle.

### Tamoxifen administration

Animals received tamoxifen injections between 9 weeks and 11 weeks of age. A solution of 15 mg ml^−1^ tamoxifen (Sigma-Aldrich, T5648) was prepared in corn oil and stored at 4 °C. Then, 1.5 mg (approximately 100 μl) of tamoxifen was delivered intraperitoneally daily for five consecutive days to each mouse. A chase period of at least 9 days was given before commencing any experimental protocols to allow for tamoxifen to be fully metabolized.

### Muscle injury

Mice were anesthetized with isoflurane, and mice were injected with 500 μl of buprenorphine (0.3 mg ml^−1^) to relieve pain. One limb was wiped with a 70% alcohol pad to sanitize the skin. Then, 100 μl of NTX solution (2 μg ml^−1^) (Accurate Chemical & Scientific, TXL8104-100) was injected into the TA/extensor digitorum longus (EDL) muscles with an insulin syringe.

### In vivo pharmacological blockade of IL-6R and Spp1

A solution of the anti-IL-6R monoclonal antibody (1 mg ml^−1^) (Bio X Cell, BE0047) and the mouse Spp1 polyclonal antibody (200 μg ml^−1^) (R&D Systems, AF808) were prepared in PBS shortly before the injections. Forty-eight hours after muscle injury, aged mice (24 months) were intramuscularly injected with 50 μl of either PBS or the antibody solution. Treatment was repeated for a total of five injections. Mice were collected at 14 dpi.

### Cell isolation by FACS

A single-cell suspension was prepared as previously described^81^. Mouse hindlimb muscles were mechanically minced and enzymatically digested with Collagenase II (1,000 U ml^−1^) for 1 hour at 37 °C in Ham’s F10 containing 10% horse serum and 1% penicillin−streptomycin. The resulting muscle slurry was further digested with a mixture of Collagenase II (1,000 U ml^−1^) and dispase (11 U ml^−1^) for an additional 30 minutes at 37 °C. The digested suspension was passed 10 times through a 20-gauge needle syringe, and the single-cell suspension was then filtered with a 40-μm cell strainer to eliminate debris. Cells were incubated with the following primary antibodies: APC anti-mouse CD31 (BioLegend, 102510; 1:100), APC anti-mouse CD45 (BioLegend, 103112; 1:100), Pacific Blue anti-mouse Ly-6A/E (anti-Sca1, BioLegend, 108120; 1:100), biotin anti-mouse CD106 (anti-VCAM1, BioLegend, 105703; 1:50), PE-Cy7 streptavidin (BioLegend, 405206; 1:100) and PE-α7-integrin (clone: R2F2, University of British Columbia, 53-0010-01; 1:500). 7-AAD (Thermo Fisher Scientific, A1310, 1:1000) was used to identify living cells. MuSCs were identified as CD31^−^CD45^−^Sca1^−^VCAM-1^+^α7-integrin^+^ events. FAPs were identified as CD31^−^CD45^−^Sca1^+^VCAM-1^−^α7-integrin^−^ events.

### In vivo EdU chasing

EdU was prepared at 5 mg ml^−1^ in PBS solution. Mice were injured with NTX, and 1 mg of EdU (approximately 200 μl) was delivered intraperitoneally to each mouse twice, at 24 hours and 12 hours before tissue harvesting. Mice were euthanized on day 4, and TA/EDL muscles were collected and cryo-embedded in O.C.T. in a methylbutane bath. Muscles were further sectioned and analyzed by immunofluorescence and confocal imaging.

### Histology and Immunofluorescence

TA/EDL muscles from uninjured or injured mice were collected and snap frozen in cold methylbutane with O.C.T. cover. Cryo-embedded tissues were mounted on a Leica cryostat and sectioned at 10-μm thickness. Cryo-sections were air dried for at least 10 minutes before being processed immediately or stored long term at −80 °C. Muscle sections were fixed with 3.7% paraformaldehyde (PFA) for 15 minutes at room temperature and rinsed with 1× PBS plus 0.3% Triton X-100 (PBST) three times. Slides were incubated for 1 hour at room temperature with a blocking buffer consisting of 1× PBST with 2% BSA and 5% of normal goat serum or normal donkey serum. Muscle sections were then incubated with primary antibodies diluted in blocking buffer for 1 hour at room temperature or overnight at 4 °C. Secondary antibodies diluted in PBST were applied for 1 hour at room temperature and rinsed with 1× PBST three times. Sections were counterstained with DAPI (Thermo Fisher Scientific, 62248) for 4 minutes and rinsed with 1× PBS three times. For collagen detection, slides were incubated with Picrosirius Red (Abcam, ab150681) as recommended by the manufacturer’s manual with minor changes. Muscle cryo-sections were air dried and hydrated in descending concentrations of ethanol. Sections were rinsed with ddH_2_O and then fixed with 4% PFA for 30 minutes at room temperature. Slides were washed with ddH_2_0 for 10 minutes, and Picrosirius Red solution (Abcam, ab150681) was applied to cover the tissue sections for 60 minutes at room temperature. Slides were rinsed in two changes of acetic acid solution and dehydrated in ascending concentrations of ethanol. Slides were soaked in xylene and mounted with Permount mounting media (Thermo Fisher Scientific, SP15-100) for brightfield microscopy imaging.

EdU Click-iT Plus Assay Kit (Abcam, ab150681) was used following the manufacturer’s recommendations. Cells or muscle sections were fixed with 4% PFA for 15 minutes and permeabilized in 0.5% Triton X-100 for 30 minutes. Samples were then washed twice in 3% BSA and incubated with Click-iT Plus reaction cocktails for 30 minutes. Samples were then washed and counterstained with DAPI to label nuclei.

To detect apoptosis, Click-iT Plus TUNEL Assay Kit (Thermo Fisher Scientific, C10618) was employed following the manufacturer’s recommendations. In brief, muscle sections were fixed in 4% PFA for 15 minutes at 37 °C and incubated with proteinase K for 5 minutes. TdT reaction mixture was applied to each slide for 60 minutes at 37 °C, followed by incubation with Click-iT Plus TUNEL reaction cocktail for 30 minutes at 37 °C. Samples were then washed and counterstained with DAPI to label nuclei.

Images were captured by a fluorescence microscope (Leica DMi8) or a confocal microscope (Leica SP8). Quantification of Pax7^+^, GFP^+^, EdU^+^, αSMA and Picrosirius Red, Pdgfrα and Tnc integrated density (int den) was performed in ImageJ 1.53 software (Fiji). Sample images were prepared in Adobe Photoshop 11.0 software.

### Myofiber size analysis

Myofiber CSA was determined by staining TA sections with Laminin, and quantification was performed with ImageJ sofware. Images were converted on 8-bit grayscale, and thresholded and particle areas were automatically calculated. The values are shown as frequency of distribution per myofiber size.

### Immunoblotting

Cells were lysed in RIPA buffer plus protein inhibitors (MilliporeSigma, 11697498001) for 15 minutes on ice, and cell lysate was centrifuged for 15 minutes at 17,000*g* at 4 °C. Extracted protein concentration was measured using Bradford assay. Protein (15 μg) was run on a 4−12% NuPAGE Bis-Tris gel (Invitrogen, NP0322BOX) and transferred onto a nitrocellulose membrane (Invitrogen, LC2001). Depending on the primary antibody, membranes were blocked with 5% milk or BSA in TBST and incubated overnight with primary antibodies at 4 °C on a rocking shaker. HRP-conjugated secondary antibodies were used to 1:5,000 dilution and visualized with Radiance Q Chemiluminescent Substrate (Azure Biosystems, AC2101) on a C600 Azure Imaging System.

### Antibodies

Antibodies used for immunofluorescence and western blot were purchased from the following companies: anti-αSMA (ab124964) was purchased from Abcam; anti-Pax7 supernatant was purchased from the Developmental Study Hybridoma Bank; anti-Pdgfrα (AF1062) and anti-OPN (AF808) were purchased from R&D Systems; anti-actin, anti-Ezh2 (3147), anti-GAPDH, anti-H3K27me3 (9733), anti-IL-6 (12912), anti-NF-κB p65 (8242), anti-Spp1 (27927) and β-tubulin (2146) were obtained from Cell Signaling Technology; and anti-Laminin (L9393), anti-Laminin (SAB4200719) and anti-Tnc (AB19013) were purchased from Sigma-Aldrich. DAPI solution (62248) was purchased from Thermo Fisher Scientific.

### Cell culture

Freshly isolated FAPs and MuSCs derived from either control or *iEzh2*^−/−^ mice were plated at a density of 15,000 cells per cm^2^ onto Collagen I-coated plates and cultured in DMEM medium (Thermo Fisher Scientific, 11965092) containing 10% FBS (Thermo Fisher Scientific, 26140079), 10% horse serum (Thermo Fisher Scientific, 16050122) supplemented with 2.5 ng ml^−1^ bFGF and 1% penicillin−streptomycin (Thermo Fisher Scientific, 15140122). Cells were allowed to attach in a 5% CO_2_ incubator and, 72 hours later, were processed for EdU assay or collected for scRNA-seq. For EdU incorporation, cells were incubated with 5 μM EdU for 30 minutes and subsequently fixed with 4% PFA. Fixed cells were washed with 1× PBS and stored at 4 °C for subsequent immunofluorescence and Click-iT EdU assay. Cells allocated for scRNA-seq were washed with PBS, detached and counted and loaded into a Chromium Next GEM chip according to the manufacturer’s recommendations. For in vitro inhibition of IL-6 and Spp1 signaling, freshly isolated FAPs and MuSCs were plated as described above and incubated with 20 μg of anti-IL-6R and 20 μg of anti-Spp1. After 48 hours, cells were incubated with 5 μM EdU for 30 minutes and subsequently fixed with 4% PFA. For cytokine detection assays, freshly isolated MuSCs were plated at a density of 35,000 cells per cm^2^ onto Collagen I-coated plates and cultured for 48 hours in DMEM medium containing 10% FBS, 10% horse serum supplemented with 2.5 ng ml^−1^ bFGF and 1% penicillin−streptomycin. Conditioned medium was collected, spun down at 5,000 r.p.m. for 5 minutes at 4 °C to remove cells and debris and stored at −80 °C.

### ELISA

ELISA was performed using MuSC-derived 48-hours conditioned media with Mouse Osteopontin (OPN/SPP1) ELISA Kit (Thermo Fisher Scientific, EMSPP1).

### Luminex assay

Luminex immunoassay was performed on supernatants deriving from MuSCs of young, i*Ezh2*^−/−^ and aged mice, cultured for 48 hours. IL-6 concentration was determined using Invitrogen mouse ProcartaPlex 8-plex Luminex immunoassay kits (Thermo Fisher Scientific, PPX-08 and EPX010-20440-901) according to the manufacturer’s protocols. The mouse cytokine standards from the assay kit were used as standards for the calculation of the cytokine concentrations. Each sample was assessed in duplicates. The differences in the cytokine concentration were statistically analyzed using the Mann−Whitney test and plotted with the GraphPad Prism software package.

### Genomic DNA extraction and quantitative real-time PCR

MuSC DNA was extracted from control and i*Ezh2*^−/−^ mice using the QIAmp DNA Micro Kit (Qiagen, 56304) according to the manufacturer’s recommendations. Genomic deletion of *Ezh2* was assessed by performing real-time PCR using Power SYBR Green Master Mix (Thermo Fisher Scientific, 25742) according to the manufacturer’s instructions. For a list of primers employed, refer to Supplementary Table 4.

### siRNA knockdown of NF-κB

Accell mouse NF-κB siRNA (Dharmacon, E-047764-00-0005) and Accell non-targeting control pool (Dharmacon, D-001910-10-05) were purchased from Dharmacon. FACS-isolated MuSCs from 3-dpi aged (24-month-old) mice were plated at a density of 35,000 cells per cm^2^ onto Collagen I-coated plates and cultured in Accell siRNA delivery medium (Dharmacon, B-005000-500). Soon after plating, Accell mouse NF-κB siRNA was added into the media at a concentration of 4 μM. Cells were cultured for 36 hours and then collected for RNA extraction. For a list of primers employed, refer to Supplementary Table 4.

### Quantitative real-time PCR

RNA was extracted using the RNeasy Micro Kit (Qiagen) and then reverse transcribed to cDNA with random primers (Invitrogen) and SuperScript III (Invitrogen), according to the manufacturer’s protocol.

### RNA-seq

RNA was extracted from freshly isolated MuSCs and FAPs at 0 dpi (uninjured), 3 dpi and 7 dpi using the RNeasy Micro Kit (Qiagen, 74004) according to the manufacturer’s instructions, and RNA was stored at −80 °C. Illumina RNA libraries were prepared with NEBNext Ultra II RNA library preparation kit for Illumina (New England Biolabs, E7775) according to the manufacturer’s instructions. In brief, 100 ng of total RNA was enriched for poly (A)^+^ mRNA and retrotranscribed. cDNAs were then fragmented, and adapters were added to each end of the fragments. The obtained libraries were amplified, and the size was selected before next-generation sequencing (NGS) analysis. All libraries were diluted to 3 nM and sequenced on an Illumina NovaSeq6000 or Illumina NextSeq550 using the following read length: 50 bp for Read1, 8 bp for I7 Index and 50 bp for Read2.

### RNA-seq analysis

Raw sequencing data were demultiplexed and converted to FASTQ with bcl2fastq/2.17.1. Reads were mapped to mouse mm10 using TopHat version 2.1.1. Reads per kilobase of transcript per million mapped reads (RPKM) values were calculated using Partek Genomics Suite 7.0. ANOVA group comparisons were performed on log_2_-transformed RPKM values using Partek GS.

### Gene set enrichment analysis

Gene set enrichment analysis (GSEA) was performed to compare transcripts upregulated in i*Ezh2*^−/−^ and aged MuSCs. For this purpose, we used a public dataset from Moiseeva et al.^23^. Counts matrix files from GSE196611 were downloaded and analyzed using DESeq2. Genes that were 2× higher in older versus young mice at 3 dpi were used to generate a gene set. We used our RNA-seq dataset to generate rank files of fold changes of injured i*Ezh2*^−/−^ versus littermate control gene expression at 3 dpi and 7 dpi. Broad GSEA 4.3.3 software, tool Run GSEAPreranked, was used. Fold changes of selected MuSC genes from aged and i*Ezh2*^−/−^ were presented on a heat map.

### scRNA-seq

scRNA-seq libraries were prepared using the Chromium Single Cell 3’ Library & Gel Bead Kit version 3.1 (10x Genomics, P/N 1000121). GEMs were generated by combining barcoded Single Cell 3’ version 3.1 Gel Beads with freshly isolated MuSCs and FAPs and partitioning oil onto Chromium Next GEM Chip G. GEM-RT was performed in a C1000 Touch Thermal Cycler with 96-Deep Well Reaction Module (Bio-Rad, P/N 1851197): 53 °C for 45 minutes, 85 °C for 5 minutes; hold at 4 °C. After retrotranscription, GEMs were broken, and the single-strand cDNA was purified with DynaBeads MyOne Silane Beads. cDNA was amplified using the C1000 Touch Thermal Cycler with 96-Deep Well Reaction Module: 98 °C for 3 minutes; cycled 11 times: 98 °C for 15 seconds, 63 °C for 20 seconds and 72 °C for 1 minute; 72 °C for 1 minute; hold at 4 °C. Amplified cDNA product was purified with the SPRIselect Reagent Kit (0.6× SPRI). Indexed sequencing libraries were constructed using the reagents in the Chromium Single Cell 3’ Library & Gel Bead Kit version 2 (10x Genomics, PN-120237). The final libraries were diluted to 3 nM and sequenced on an Illumina NovaSeq6000 using the following read length: 28 bp for Read1, 8 bp for I7 Index and 91 bp for Read2.

### scRNA-seq analysis

The 10x Genomics Cell Ranger version 7.0.0 analysis pipeline was used to generate FASTQ files and count matrices using default parameters with the 10x Genomics mm10 reference (refdata-gex-mm10-2020-A). Single-cell quality control filtering and analysis were performed using Seurat version 4.3.0 (ref. ^82^) in R version 4.1.1 (ref. ^83^). Cells with a low gene count (<500 genes), a high gene count (>6,500 genes for control FAPs 7 dpi, >6,000 for others), a high transcript count (>70,000) and a high mitochondrial percentage (>5%) were filtered out to remove low-quality, potential doublets and dying cells. Dimensional reduction of the merged log_10-_normalized data was performed using 50 principal components and visualized using UMAP. For scRNA-seq on *PDGFRα*^*EGFP*^ FAPs, unsupervised clustering was performed with a resolution of 0.6. Clusters with fewer than 50 cells (clusters 11−13) were removed, and cluster 14 was merged with cluster 7, based on shared cell markers. Top markers for each cluster were calculated using the Wilcoxon rank-sum test. Split violin plots were created with custom scripts using ggplot2 version 3.5.1 (ref. ^84^). For scRNA-seq in co-culture MuSCs and FAPs, unsupervised clustering was performed with a resolution of 0.3, and clusters 6 and 7 were removed because of low cell counts. The top markers for each cluster were calculated using the Wilcoxon rank-sum test. Based on FAP and MuSC markers, Seurat clusters 0 and 5 were merged to create the FAPs cluster; Seurat clusters 1 and 4 were merged to create the activated MuSCs cluster; and Seurat clusters 2 and 3 were merged to create the differentiated MuSCs cluster.

### CellChat analysis

Cell communication was analyzed using CellChat version 1.6.1 (ref. ^49^). The CellChat function ranknet (parameters: mode = ‘comparison’, stacked = F, do.stat = TRUE) was used to generate the data for Fig. 4d, and the bar plot was created using ggplot2 version 3.5.1 (ref. ^84^).

### CUT&RUN

CUT&RUN was conducted using the CUT&RUN assay kit (Cell Signaling Technology, 86652), following the manufacturer’s instructions. MuSCs were crosslinked with 0.1% formaldehyde for 2 minutes at room temperature. For each reaction, 130,000 MuSCs were gently resuspended in 100 µl of the supplied wash buffer, and 30,000 MuSCs were used as input. MuSCs were bound to Concanavalin- A beads and incubated overnight with the following antibodies: anti-IgG (Cell Signaling Technology, 2729), anti-H3K27me3 (Cell Signaling Technology, 9733) or anti-NF-κB (Cell Signaling Technology, 8242). The enzyme pAG-MNase was added to the suspension, incubated at 4 °C for 1 hour and then activated by adding 3 μl of cold calcium chloride to each tube and rotated at 4 °C for 30 more minutes. Samples were resuspended with the STOP buffer provided by the kit and kept at 37 °C for 10 minutes to release the DNA fragments into the solution. The final DNA was isolated using spin columns and resuspended to 3 nM for NGS library generation. The final libraries were diluted to 3 nM and sequenced on an Illumina NextSeq 2000 using the following setting: R1: 50 cycles, R2: 50 cycles and i7: eight cycles.

To evaluate NF-κB recruitment at the *IL6* and *Spp1* loci, the isolated DNA was subjected to qPCR amplification. For a list of primers employed for qPCR, refer to Supplementary Table 4.

### CUT&RUN data processing and analysis

FASTQ files were generated with bcl2fastq/2.20.0. Paired-end reads were processed using CutRunTools/20200629 (ref. ^85^) with minor modifications. In brief, adapter sequences and low-quality bases were trimmed using Trimmomatic/0.39 (ref. ^86^). Trimmed reads were aligned to mm10 mouse genome using Bowtie/2-2.5.3 (ref. ^87^). Duplicate reads were marked using Picard/2.27.3 (http://broadinstitute.github.io/picard/) and removed using SAMtools/1.9 (ref. ^88^) with flags -bh -F 1024. H3K27me3 peaks were called by MACS2 (ref. ^89^) with the following parameters: –broad–broad-cutoff 0.1 -B–SPMR–keep-dup all. For visualization, BAM files were first converted to bedGraph format using BEDTools and then to BigWig format using the UCSC bedGraphToBigWig tool. Signal tracks were further processed with deepTools/3.5.6 (ref. ^90^), where the BigWig files were corrected against IgG control using the bigwigCompare function with the –operation log_2_ parameter. TSS enrichment profiles were generated to compare the H3K27me3 signal distribution between adult and aged MuSCs.

### Statistics and reproducibility

Statistical analysis was performed using GraphPad Prism 10.2.3 software. All statistical data show the mean ± s.d. of at least three biologically independent experiments or samples. Unpaired, two-tailed *t*-test, one-way ANOVA with Dunnett’s multiple comparison test and two-way ANOVA with Sidak’s multiple comparison were performed in GraphPad Prism. *P* values are provided in the corresponding figures. The number of independent experiments or quantified regions is listed in the corresponding figure legends. No statistical methods were used to predetermine sample sizes. Experimental blinding was used, and no experimental data were excluded. Data distribution was assumed to be normal, but this was not formally tested.

### Reporting summary

Further information on research design is available in the Nature Portfolio Reporting Summary linked to this article.

## Supplementary information

Reporting Summary

Supplementary Tables 1−4 Senescence-associated transcripts in uninjured control and iEzh2^−/−^ MuSCs; scRNA-seq FAP 7 dpi; Genes modulated in iEzh2^−/−^ and aged MuSCs; DNA oligonucletotides.

## Source data

Source Data Fig. 1 Statistical source data.

Source Data Fig. 2 Statistical source data.

Source Data Fig. 3 Statistical source data.

Source Data Fig. 4 Statistical source data.

Source Data Fig. 5 Statistical source data.

Source Data Fig. 6 Statistical source data.

Source Data Fig. 7 Statistical source data.

Source Data Fig. 8 Statistical source data.

Source Data Extended Data Fig. 1 Statistical source data.

Source Data Extended Data Fig. 2 Statistical source data.

Source Data Extended Data Fig. 3 Statistical source data.

Source Data Extended Data Figs. 1–7 Unprocessed western blots.

## Data Availability

The sequencing data generated in this study have been deposited in the Gene Expression Omnibus under accession code GSE282342. This paper does not report original code. Any additional information required to reanalyze the data reported in this paper is available from the lead contact upon reasonable request. Uncropped and unprocessed immunoblots are available in the Source Data file. Source data are provided with this paper.
