# Supplementary figures and images for: Epigenetic dysregulation in aged muscle stem cells drives mesenchymal progenitor expansion via IL-6 and Spp1 signaling
Source: Nat Aging. 2025 Oct 29;5(12):2399–416. doi: 10.1038/s43587-025-01002-0 (PMC12705444; doi:10.1038/s43587-025-01002-0)

C

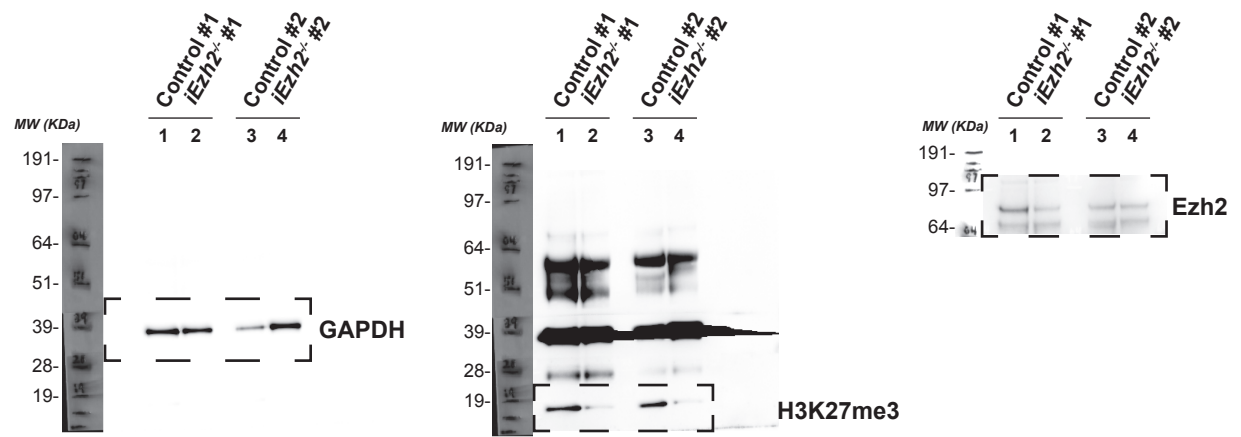

**b**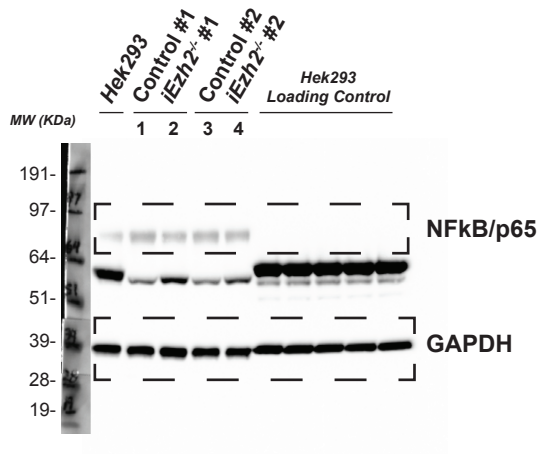**d**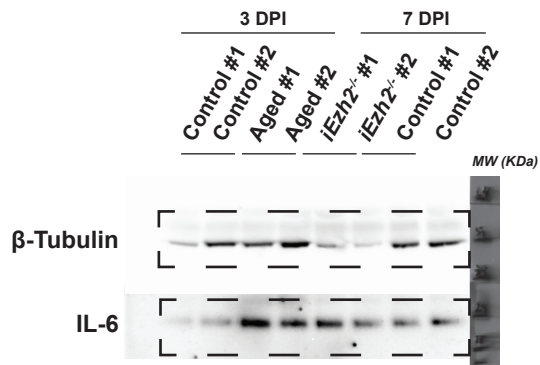**e**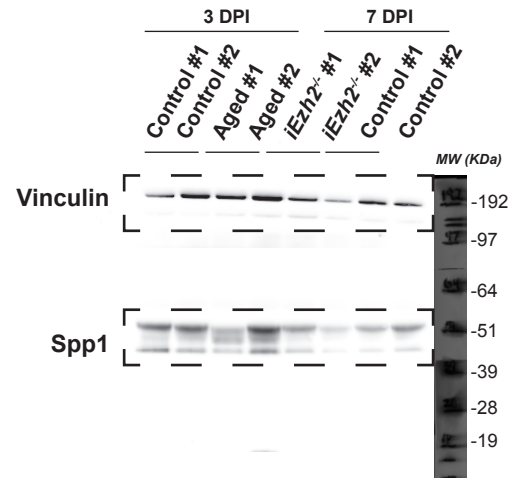

Supplement: Supplementary file 14 — Unprocessed western blots. [file 43587_2025_1002_MOESM14_ESM.pdf]
